# Emotional Processing Following Digital Cognitive Behavioral Therapy for Insomnia in People With Depressive Symptoms: A Randomized Clinical Trial

**DOI:** 10.1001/jamanetworkopen.2024.61502

**Published:** 2025-02-27

**Authors:** Sandra Tamm, Katrina Y. K. Tse, Jennifer Hellier, Kate E. A. Saunders, Catherine J. Harmer, Colin A. Espie, Matthew Reid, Simon D. Kyle

**Affiliations:** 1 Centre for Psychiatry Research, Department of Clinical Neuroscience, Karolinska Institutet, Stockholm Health Care Services, Region Stockholm, Sweden; 2 Department of Psychiatry, University of Oxford, Oxford Health NHS Foundation Trust, Warneford Hospital, Oxford, UK; 3 Sir Jules Thorn Sleep and Circadian Neuroscience Institute, Nuffield Department of Clinical Neurosciences, University of Oxford, Oxford, UK; 4 Department of Biostatistics and Health Informatics, King’s College London, Institute of Psychiatry, Psychology and Neuroscience, London, UK; 5 Big Health Inc, London, UK; 6 Department of Psychiatry and Behavioral Sciences, Johns Hopkins School of Medicine, Baltimore, Maryland

## Abstract

**Importance:**

Cognitive behavioral therapy for insomnia (CBT-I) has been shown to reduce depressive symptoms, but the underlying mechanisms are not well understood and warrant further examination.

**Objective:**

To investigate whether CBT-I modifies negative bias in the perception of emotional facial expressions and whether such changes mediate improvement in depressive symptoms.

**Design, Setting, and Participants:**

A randomized clinical trial of digital CBT-I vs sleep hygiene education was conducted. Adults living in the UK who met diagnostic criteria for insomnia disorder and Patient Health Questionnaire-9 criteria (score ≥10) for depression were recruited online from the community and randomly assigned to either a 6-session digital CBT-I program or a sleep hygiene webpage. Participant recruitment took place between April 26, 2021, and January 24, 2022, and outcomes were assessed at 5 and 10 weeks post randomization. Data analysis was performed from December 1, 2022, to March 1, 2023.

**Main Outcomes and Measures:**

Coprimary outcomes were recognition accuracy (percentage) of happy and sad facial expressions at 10 weeks assessed with the facial expression recognition task. Secondary outcomes were self-reported measures of insomnia, depressive symptoms, affect, emotional regulation difficulties, worry, perseverative thinking, midpoint of sleep, social jet lag, and the categorization of and recognition memory for emotional words. Intention-to-treat analysis was used.

**Results:**

A total of 205 participants were randomly assigned to CBT-I (n = 101) or sleep hygiene education (n = 104). The sample had a mean (SD) age of 49.3 (10.1) years and was predominately female (165 [80.8%]). Retention was 85.7% (n = 175). At 10 weeks, the estimated adjusted mean difference for recognition accuracy was 3.01 (97.5% CI, −1.67 to 7.69; *P* = .15; Cohen *d* = 0.24) for happy facial expressions and −0.54 (97.5% CI, −3.92 to 2.84; *P* = .72; Cohen *d* = −0.05) for sad facial expressions. At 10 weeks, CBT-I compared with control decreased insomnia severity (adjusted difference, −4.27; 95% CI, −5.67 to −2.87), depressive symptoms (adjusted difference, −3.91; 95% CI, −5.20 to −2.62), negative affect (adjusted difference, −2.75; 95% CI, −4.58 to −0.92), emotional regulation difficulties (adjusted difference, −5.96; 95% CI, −10.61 to −1.31), worry (adjusted difference, −8.07; 95% CI, −11.81 to −4.33), and perseverative thinking (adjusted difference, −4.21; 95% CI, −7.03 to −1.39) and increased positive affect (adjusted difference, 4.99; 95% CI, 3.13-6.85). Improvement in negative affect, emotional regulation difficulties, and worry at week 5 mediated the effect of CBT-I on depression severity at 10 weeks (% mediated: 21.9% Emotion regulation difficulties; 24.4% Worry; and 29.7% Negative affect). No serious adverse events were reported to the trial team.

**Conclusions and Relevance:**

This randomized clinical trial did not find evidence that CBT-I engenders change in the perception of facial expressions at post treatment, despite improvements in insomnia and depressive symptoms. Early change in negative affect, emotional regulation difficulties, and worry mediated lagged depression outcomes and deserve further empirical scrutiny.

**Trial Registration:**

isrctn.org Identifier: ISRCTN17117237

## Key Points

**Question:**

Does cognitive behavioral therapy for insomnia modify the perception of emotional facial expressions in people with clinically significant depressive symptoms?

**Findings:**

In a randomized clinical trial that included 205 people, cognitive behavioral therapy for insomnia did not significantly change the perception of happy or sad facial expressions following treatment. However, large improvements in insomnia and depression symptoms occurred.

**Meaning:**

The findings of this trial suggest that emotion processing bias indexed by perception of facial expressions may not be an important explanatory factor in the antidepressant effects of cognitive behavioral therapy for insomnia.

## Teaser

This randomized clinical trial examines the potential impact of cognitive behavioral therapy for insomnia on putative pathways linking insomnia and depression.

## Introduction

Sleep disruption features in the diagnostic criteria for depression and low mood is a common daytime consequence of insomnia. Findings from mendelian randomization studies suggest that insomnia-related genetic variants may have a role in the manifestation of depression,^1^ and prospective studies observed that insomnia is a risk factor associated with both first-onset and recurrent depression.^2,3^

Meta-analyses report that improving sleep through cognitive behavioral therapy, the first-line recommended insomnia treatment, was associated with reductions in depressive symptoms in clinical and nonclinical populations^4,5^ and may even reduce the incidence of first-episode depression.^6^ There is also evidence that early reduction in insomnia mediates improvement in depressive symptoms at post treatment,^7^ and several hypotheses have been offered to explain how insomnia may be linked with depression.^6,8,9,10^

Emotion processing bias reflected in altered perception, attention, and memory for emotionally salient information^11^ is one plausible pathway linking insomnia and depression.^10,12^ Specifically, information processing biases assessed with behavioral tasks reveal that people with depression are less accurate in identifying happy facial expressions,^13^ attend to negative information,^14^ and recall more negative memories of themselves.^15^ This negative bias has been implicated in the development and maintenance of depression and may reflect alterations in neural plasticity and functional neuroanatomy involved in the generation and regulation of emotion.^12^ Improvement in correctly identifying happy facial expressions has been shown to be an early marker of antidepressant action^16,17,18^ and subsequent mood improvement.^17^ There is also preliminary evidence that behavioral activation, a component of cognitive behavioral therapy (CBT) for depression, may be associated with reduced recognition of negative facial expressions at post treatment compared with control.^19^

Sleep plays a key role in emotional homeostasis and, while findings are inconsistent, both experimental sleep loss^20,21^ and insomnia^22,23^ may be characterized by alterations in the appraisal of and memory for emotional stimuli, in addition to reduced positive affect,^24,25^ increased negative affect,^24^ and poorer emotion regulation.^24^ To our knowledge, no published study has been designed to specifically assess mechanisms through which insomnia treatment improves depression. We performed an explanatory randomized clinical trial to examine the potential impact of CBT for insomnia (CBT-I) on putative pathways linking insomnia and depression. We focused primarily on emotion processing and specifically on recognition of sad and happy facial expressions using a task sensitive to depression and its pharmacologic and behavioral treatment.^26^ Secondary outcomes and putative mediators of interest were emotion bias on a word categorization and memory recognition task, positive and negative affect, worry, perseverative negative thinking, emotional regulation difficulties, midpoint of sleep, and social jet lag.

## Methods

### Study Design

The Emotional Processing in Insomnia Co-Occurring With Low Mood (EPIC) study was an online parallel-group explanatory randomized clinical trial of digital CBT-I vs sleep hygiene education (SHE). Individuals meeting *Diagnostic and Statistical Manual of Mental Disorders, Fifth Edition* (*DSM-5*) criteria for insomnia disorder and reporting clinically relevant depressive symptoms were recruited from the community and randomized to CBT-I or SHE. Assessments took place online at 0 (baseline), 5 (midtreatment), and 10 (posttreatment) weeks. The study was conducted in the UK and approved by the University of Oxford Medical Sciences Interdivisional Research Ethics Committee and the trial protocol (Supplement 1) was uploaded to the Open Science Framework.^27^ This study followed the Consolidated Standards of Reporting Trials (CONSORT) reporting guideline.

### Participants

Participants were recruited from the community, including through advertising on social media platforms and university webpages. Inclusion criteria were (1) age 25 to 65 years, (2) meet *DSM-5* criteria for insomnia disorder according to the Sleep Condition Indicator,^28^ (3) report depressive symptoms in the clinically significant range (Patient Health Questionnaire-9 [PHQ-9] ≥10),^29^ (4) have access to a phone and a computer with reliable internet access, (5) read and understand English, and (6) currently live in the UK. Exclusion criteria were (1) screen positive for or report diagnosis of additional sleep disorder; (2) report suicidal thoughts or history of recent suicide attempt; (3) report habitual night, evening, or rotational shift work in the past month; (4) report psychiatric comorbidities of psychosis or bipolar disorder; (5) report a diagnosis of mild cognitive impairment, dementia, or neurologic condition; (6) current engagement in psychotherapy for insomnia or depression; (7) previous participation in online sleep treatment; (8) current use of hypnotic, psychotropic, or antiepileptic medications; (9) alcohol misuse and dependency or recreational drug use; (10) psychiatric hospital admission or contact with crisis team in the past year; and (11) serious physical health concerns necessitating surgery or with a survival prognosis of less than 6 months. Ethnicity data were collected by self-report questionnaire (presented as an ethnicity background choice on the questionnaire).

### Randomization and Blinding

Randomization was carried out online,^30^ independent of the study team, with a 1:1 allocation ratio minimizing for sex (male, female), age (25-44, 45-65 years), depressive symptom severity (PHQ-9 scores ≤15, ≥16), and insomnia severity (Insomnia Severity Index [ISI] scores ≤19, ≥20^31^) to ensure these factors were balanced across arms. Following randomization, the study team emailed participants their allocation and relevant link to access the intervention.

In all participant-facing information, the study was presented as comparing 2 interventions. The trial team were not blinded to group allocation, but all emails and phone calls were scripted, meaning participants from both trial arms received the same information. All outcomes were self-completed using either performance-based computerized tasks or questionnaires. Assessments took place online, separate from the platforms used to deliver the interventions. Analyses were performed with investigators blinded to intervention group.

### Procedures

Individuals were screened and provided informed consent using an online platform (Qualtrics). An introductory phone call was scheduled with eligible participants to provide the opportunity for them to ask questions. After completing the baseline assessment, participants were randomized to CBT-I or SHE. Assessments took place on an electronic patient-reported outcome platform (ePRO) at 0 (baseline), 5 (midtreatment), and 10 (posttreatment) weeks. At 2 weeks post randomization, a follow-up phone call was scheduled to ensure participants could access their allocated treatment.

Participants randomized to CBT-I received access to a fully automated and interactive digital CBT-I program (Sleepio). The program is based on CBT-I manuals^32,33^ and its efficacy for insomnia has been established in randomized clinical trials.^34,35,36^ Treatment was delivered by an animated therapist over six 15- to 20-minute sessions (eTable 14 and eTable 15 in Supplement 2 provide treatment content).

Participants in the control group received access to a sleep hygiene education webpage, which was based on established advice^37^ and comprised guidance on lifestyle and environmental factors associated with sleep. For both arms, there was no restriction on accessing treatments for sleep and/or mental health once enrolled in the trial, but we monitored use at postrandomization assessments. On completion of the study (10 weeks), participants in the SHE group were offered access to the digital CBT-I program. Participants received gift vouchers for completing each post-randomization assessment (£10 for mid-treatment, £15 for post-treatment). On completion of the study (10 weeks), participants in the SHE group were offered access to the digital CBT-I program.

### Outcomes

The coprimary outcomes were (1) recognition accuracy of happy facial expressions and (2) recognition accuracy of sad facial expressions at week 10 measured with the Facial Expression Recognition Task (FERT; Oxford Emotional Test Battery). The FERT assessed recognition of facial expressions featuring 6 basic emotions: happy, fear, anger, disgust, sad, and surprise. Each emotion was digitally combined with a neutral face to create 10 emotional intensities (ranging from 0% to 100%, in 10% steps). During the task, a face would appear in the center of the screen for 500 milliseconds. Participants indicated the expression of the face by clicking the corresponding button on the screen. There were 250 trials in total (10 neutral faces plus 40 faces for each emotion [4 faces for each emotional intensity]). Different sets of faces were presented at the different time points to minimize practice effects. We hypothesized that treatment compared with control would lead to increased recognition for happy faces and decreased recognition for sad faces.

Secondary outcomes were insomnia severity (ISI^31^), depression severity (PHQ-9^29^), positive and negative affect (Positive and Negative Affect Schedule [PANAS-SF]^38^), emotional regulation difficulties (Difficulties in Emotion Regulation Scale [DERS]^39^), worry (Penn State Worry Questionnaire [PSWQ-Past Week]^40^), perseverative thinking (Perseverative Thinking Questionnaire [PTQ]^41^), midpoint of sleep on work vs free days-sleep corrected, and social jet lag (Munich Chronotype Questionnaire [MCTQ]^42^). We dichotomized the ISI and PHQ-9 scales to quantify participants scoring below clinical thresholds for insomnia (ISI <11) and depression (PHQ-9 < 10) at 5 and 10 weeks.

The emotion categorization task (ECAT) assessed response speed to positive and negative self-referential personality descriptors.^43^ Participants were asked to indicate whether they would like or dislike being described according to the personality descriptor. There were 60 trials in total (30 positive and 30 negative) (outcome: reaction time [milliseconds]). The emotional recognition memory task (EMEM) assessed recognition memory for emotional words previously presented in the ECAT task plus a matching set of distracter words. Participants were asked to indicate whether the word had previously been presented (yes or no). There were 120 trials in total (60 words from ECAT [30 positive, 30 negative] plus 60 distracter words [30 positive, 30 negative]) (outcome: percentage of correctly recognized words).

We assessed whether depression score at 10 weeks was mediated by the following variables at 5 weeks: facial recognition accuracy (FERT accuracy of sad and happy expressions), categorization of emotional words (ECAT reaction time), emotional regulation difficulties (DERS), worry (PSWQ), preservative thinking (PTQ), midpoint of sleep (MCTQ), and positive and negative affect (PANAS). To provide context to trial findings, we descriptively report 4 outcomes that were not preregistered hypotheses: use of sleep medications, engagement with psychological therapy for sleep problems, receipt of treatment for mental health, and sudden mood changes.

Serious adverse events were defined as death, suicide attempt, and admissions to secure psychiatric units. Serious adverse events were recorded if the study team was informed by participants of such events. Suicidal ideation was assessed using item 9 (score >0) of the PHQ-9 at baseline, 5 weeks, and 10 weeks.

### Statistical Analysis

Data analysis was performed from December 1, 2022, to March 1, 2023. We calculated that 100 participants per treatment group were needed to detect a standardized effect size of 0.5 on the FERT at 90% power and 5% significance level, accounting for an estimated attrition rate of 15%. Our primary intention-to-treat analyses followed the agreed statistical analysis plan (Supplement 1), finalized before completion of data collection and inspection of postrandomization data. Analyses were done using R, version 4.2.2 (R Foundation for Statistical Computing). All participants who were randomized and had at least one outcome measure were included in the analyses. Participants who withdrew were included in the analyses until the point they withdrew. Participants were analyzed according to their allocated treatment group irrespective of what treatment they actually received. Data preprocessing steps can be found in the eMethods in Supplement 2.

Each primary and secondary continuous outcome measure was analyzed using a linear mixed-effects model, with the outcome measurement (at the two follow-up time points) as the dependent variable. The models included fixed effects for the baseline measure of the outcome (assuming a linear relationship between baseline and outcome), the randomization variables (sex, age, baseline ISI score, and baseline PHQ-9 score), treatment arm (CBT-I or SHE), and assessment time point (5 or 10 weeks). An interaction between time point and treatment arm was also included. For continuous outcome variables, modeling was based on a normal distribution. The dichotomized outcomes were analyzed using generalized linear mixed-effect models with a logit link function.^44^ All models included a random intercept for participant where the variance-covariance structure was assumed to be unstructured and were fitted using restricted maximum likelihood estimation. The primary analysis assumed data were missing at random.

Treatment effect estimates are presented as the adjusted mean difference between groups, with 97.5% CIs and *P* values for primary analysis (coprimary outcomes of FERT recognition accuracy for happy and sad faces at week 10) and 95% CIs and *P* values for all other analyses. Coprimary outcomes were considered statistically significant at *P*<.025, while all secondary outcomes were considered significant at *P* < .05. Cohen *d* effect sizes were calculated by dividing the adjusted between-group difference by the overall group baseline SD of the corresponding outcome.

We performed 2 prespecified sensitivity analyses to investigate the robustness of primary outcome findings to assumptions regarding outcome missingness: adjustment for baseline variables associated with outcome missingness and multiple imputation. A complier-average causal effect (CACE) analysis was carried out using the ivreg package in R^45^ to determine the effect of intervention adherence on primary outcomes at 10 weeks. Adherence was defined as attending at least 3 sessions of the digital CBT-I program; the 2-stage least-squares approach was used to estimate the instrumental variable.^46^ CACE models were adjusted for baseline characteristics associated with treatment adherence, baseline value of the outcome, and randomization variables.

We did a prespecified exploratory subgroup analysis of the primary outcome by baseline depression severity (PHQ-9), worry (PSWQ), emotional regulation difficulties (DERS), and midpoint of sleep (MCTQ, midpoint of sleep on free days, adjusted for sleep debt). We added a 3-way interaction term between randomized group, time point, and subgroup variable to estimate the treatment effects at each time point and in each subgroup.

Parametric regression models tested for mediation of CBT-I on PHQ-9 outcomes through potential mediators. Mediation analyses were conducted using the mediation package in R.^47^ Listwise deletion was applied to missing data.^48^ Analyses were adjusted for baseline measures of the mediator and randomization variables.

## Results

### Screening and Baseline Characteristics 

**Figure 1.**
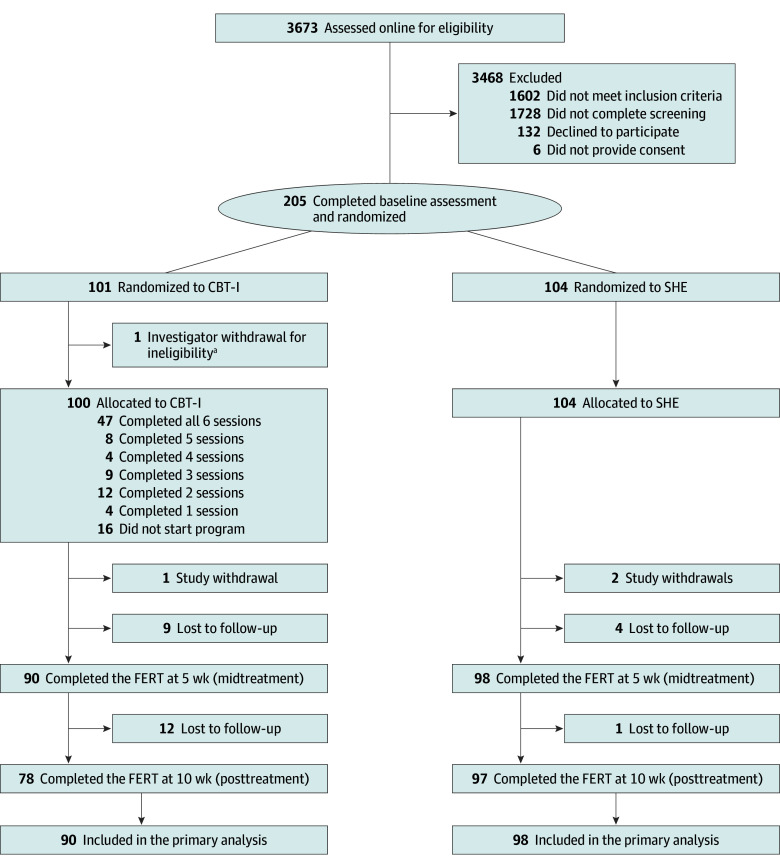
Trial Design CBT-I indicates cognitive behavioral therapy for insomnia; FERT, facial expression recognition task; and SHE, sleep hygiene education. ^a^This was treated similar to a screening exclusion, such that any research data they provided were not used in the analysis.

Recruitment started on April 26, 2021, and ended on January 24, 2022, when the recruitment target was reached. The eligibility questionnaire was completed by 3673 individuals, of whom 337 were deemed eligible. A total of 205 participants completed the baseline questionnaire and were subsequently randomized to either CBT-I (n = 101) or SHE (n = 104) (Figure 1). The main reasons for exclusion were not meeting insomnia criteria, responses indicative of possible sleep disorder other than insomnia, and shift work (eTable 1 in Supplement 2).

The sample was predominantly female (165 [80.8%]), with a mean (SD) age of 49.3 (10.1) years, primarily White ethnicity background (based on the UK census categories) (187 [91.6%]), employed full-time or part-time (154 [75.5%]), and university educated (151 [74.0%]) (Table 1). Consistent with inclusion criteria, ISI scores (mean [SD], 19.25 [3.66]) and PHQ-9 scores (mean [SD], 14.25 [4.25]) were in the clinical range. Groups were generally well matched on demographic and clinical outcomes at baseline (Table 1).

**Table 1.** Sociodemographic and Clinical Characteristics of the Participants

Characteristic	No. (%)		
	CBT-I^a^ (n = 100)	SHE (n = 104)	Overall (N = 204)
**Demographic characteristic**			
Age, y	49.7 (10.2)	48.8 (10.0)	49.3 (10.1)
Sex			
Female	80 (80.0)	85 (81.7)	165 (80.9)
Male	19 (19.0)	19 (18.3)	38 (18.6)
Prefer not to say	1 (1.0)	0 (0.0)	1 (0.5)
Ethnicity^b^			
Asian or Asian British or Asian Scottish	4 (4.0)	2 (1.9)	6 (3.0)
Caribbean or Black	1 (1.0)	0	1 (0.5)
Mixed/multiple ethnic	1 (1.0)	4 (3.8)	5 (2.4)
White	90 (90.0)	97 (93.3)	187 (91.6)
Other	3 (3.0)	1 (1.0)	4 (2.0)
Prefer not to say	1 (1.0)	0	1 (0.5)
Employment status			
Full-time	39 (39.0)	47 (45.2)	86 (42.2)
Part-time	32 (32.0)	36 (34.6)	68 (33.3)
Unemployed	6 (6.0)	5 (4.8)	11 (5.4)
Retired	13 (13.0)	13 (12.5)	26 (12.7)
Full-time student	3 (3.0)	1 (1.0)	4 (2.0)
Full-time homemaker or carer	7 (7.0)	2 (1.9)	9 (4.4)
Highest level of qualification			
GCSE or equivalent	6 (6.0)	7 (6.7)	13 (6.4)
A level or equivalent	6 (6.0)	6 (5.8)	12 (5.9)
College	13 (13.0)	15 (14.4)	28 (13.8)
University undergraduate	44 (44.0)	41 (39.4)	85 (41.7)
University postgraduate	31 (31.0)	35 (33.7)	66 (32.5)
**Baseline scores**			
FERT, mean (SD)			
Accuracy: happy faces, %	46.89 (12.68)	47.73 (12.16)	47.32 (12.40)
Accuracy: sad faces, %	68.25 (10.97)	71.49 (10.78)	69.90 (10.97)
Insomnia severity, ISI, mean (SD)	19.34 (3.52)	19.15 (3.81)	19.25 (3.66)
Sleep quality, PSQI, mean (SD)			
TST, min	303.30 (62.83)	313.13 (77.24)	308.31 (70.55)
SOL, min	67.45 (52.98)	61.54 (43.68)	64.44 (48.43)
Sleep efficiency, %	63.49 (23.12)	62.72 (18.16)	63.10 (20.69)
Depression severity, PHQ-9, mean (SD)	13.79 (3.96)	14.68 (4.50)	14.25 (4.25)
Positive and negative affect, PANAS, mean (SD)			
Positive affect	21.82 (7.42)	20.41 (6.26)	21.10 (6.87)
Negative affect	25.25 (7.37)	26.61 (7.57)	25.94 (7.49)
Emotional regulation difficulties, DERS, mean (SD)	94.73 (22.52)	100.61 (22.25)	97.73 (22.52)
Worry, PSWQ, mean (SD)	60.66 (15.33)	63.88 (13.22)	62.30 (14.35)
Perseverative Thinking, PTQ, mean (SD)	36.95 (9.52)	39.77 (9.21)	38.39 (9.45)
Chronotype, MCTQ^c^			
Midpoint of sleep period on work-free days, sleep corrected, HH-MM, mean (SD) [No.]	03:14 (76 [73])	03:26 (81 [76])	03:20 (79 [149])
Social jet lag, min, mean (SD) [No.]	35.17 (39.44) [93]	31.86 (34.38) [91]	33.54 (36.96) [184]
Emotional categorization task, ECAT, mean (SD)			
Reaction time: positive words, ms	1463.79 (220.03)	1429.85 (210.81)	1446.49 (215.52)
Reaction time: negative words, ms	1474.17 (221.45)	1451.32 (211.65)	1462.52 (216.28)
EMEM, mean (SD)			
Accuracy: positive words, %	75.28 (10.14)	73.32 (11.83)	74.28 (11.05)
Accuracy: negative words, %	77.00 (10.51)	75.29 (12.30)	76.13 (11.46)

Abbreviations: CBT-I, Cognitive Behavioral Therapy for Insomnia; DERS, Difficulties in Emotional Regulation Scale; EMEM, Emotion Recognition Memory Task; FERT, Facial Expression Recognition Task; GCSE, General Certification for Secondary Education; HH:MM, clock time in hours and minutes format; ISI, Insomnia Severity Index; MCTQ, Munich Chronotype Questionnaire; PANAS, Positive and Negative Affect Schedule; PHQ-9, Patient Health Questionnaire-9; PSQI, Pittsburgh Sleep Quality Index; PSWQ, Penn State Worry Questionnaire; PTQ, Perseverative Thinking Questionnaire; SHE, Sleep Hygiene Education; SOL, sleep onset latency; TST, total sleep time.

^a^ The N for CBT-I reflects number following removal of 1 participant due to ineligibility. This was treated similar to a screening exclusion, such that any research data they provided were not used in the analysis.

^b^ Ethnicity data were collected by self-report questionnaire. The ethnic group categories were based on the UK census categories.

^c^ Bracketed values represent different numbers of data contributed to MCTQ variables.

Retention was 92.2% (n = 188) at 5 weeks and 85.7% (n = 175) at 10 weeks. Dropout from study assessments was greater in the CBT-I group (22 [21.8%]) than the SHE group (7 [6.7%]) (eTable 4 in Supplement 2). Compared with participants who provided primary outcome data at 10 weeks, participants who had missing data scored significantly higher on baseline positive affect (eTable 5 in Supplement 2). In the CBT-I group, 47.0% (n = 47) of the participants completed all 6 online treatment sessions, 68.0% (n = 68) completed at least 3 sessions (considered treatment adherence), and 16.0% (n = 16) did not access the intervention (eTable 7, eTable 8 in Supplement 2). There were 4 postrandomization withdrawals from the study (2 in CBT-I and 2 in SHE) (eTable 2 in Supplement 2).

### Primary and Secondary Outcomes 

Large treatment effects were observed for reduction in insomnia severity (ISI) at both 5 weeks (adjusted difference, −3.42; 95% CI, −4.79 to −2.05; Cohen *d* = −0.93) and 10 weeks (adjusted difference, −4.27; 95% CI, −5.67 to −2.87; Cohen *d* = −1.16), with the CBT-I group reporting fewer insomnia symptoms (Table 2). Medium to large treatment effects in favor of CBT-I were also observed for depression symptom severity (PHQ-9) at both 5 weeks (adjusted difference, −2.49; 95% CI, −3.75 to −1.23; Cohen *d* = −0.59) and 10 weeks (adjusted difference, −3.91; 95% CI, −5.20 to −2.62; Cohen *d* = −0.92). The CBT-I intervention was associated with a higher likelihood of scoring below clinical thresholds for both depression (PHQ-9 <10) and insomnia (ISI <11) compared with SHE at 5 and 10 weeks (eTable 3 in Supplement 2).

**Table 2.** Effects of CBT-I vs SHE on Primary and Secondary Outcomes

Assessment	Unadjusted				Adjusted difference (95% CI)^a^	*P* value	Cohen *d* (95% CI)
	CBT-I		SHE				
	Mean (SD)	No.	Mean (SD)	No.			
**FERT accuracy: happy faces, %**							
Week 5	54.72 (16.53)	88	50.72 (16.54)	98	4.53 (0.53 to 8.53)	.03	0.37 (0.04 to 0.69)
Week 10^b^	53.44 (16.77)	78	51.39 (15.75)	97	3.01 (−1.67 to 7.69)^b^	.15	0.24 (−0.09 to 0.58)
**FERT, accuracy: sad faces, %**							
Week 5	64.90 (11.22)	88	67.02 (9.71)	98	−0.50 (−3.40 to 2.40)	.74	−0.05 (−0.31 to 0.22)
Week 10^b^	61.18 (13.77)	78	63.35 (11.17)	97	−0.54 (−3.92 to 2.84)^b^	.72	−0.05 (−0.32 to 0.22)
**Insomnia severity, ISI, total scores**							
Week 5	12.93 (4.78)	88	16.36 (5.25)	98	−3.42 (−4.79 to −2.05)	<.001	−0.93 (−1.30 to −0.56)
Week 10	11.13 (5.53)	78	15.59 (5.40)	97	−4.27 (−5.67 to −2.87)	<.001	−1.16 (−1.54 to −0.78)
**Depression severity, PHQ-9, total scores**							
Week 5	9.49 (5.10)	88	12.60 (5.82)	98	−2.49 (−3.75 to −1.23)	<.001	−0.59 (−0.88 to −0.29)
Week 10	7.90 (4.95)	78	12.35 (5.54)	97	−3.91 (−5.20 to −2.62)	<.001	−0.92 (−1.22 to −0.62)
**Affect, PANAS, positive**							
Week 5	24.86 (8.83)	88	22.16 (6.61)	98	1.88 (0.05 to 3.71)	.045	0.27 (0.01 to 0.54)
Week 10	26.86 (8.27)	78	21.48 (6.53)	97	4.99 (3.13 to 6.85)	<.001	0.73 (0.45 to 1.00)
**Affect, PANAS, negative **							
Week 5	20.94 (7.67)	88	25.76 (8.47)	98	−3.63 (−5.42 to −1.84)	.001	−0.48 (−0.72 to −0.25)
Week 10	20.37 (7.79)	78	24.26 (8.55)	97	−2.75 (−4.58 to −0.92)	.004	−0.37 (−0.61 to −0.12)
**Emotional regulation difficulties, DERS, total scores**							
Week 5	84.93 (22.17)	88	95.90 (22.22)	98	−6.04 (−10.61 to −1.47)	.01	−0.27 (−0.47 to −0.07)
Week 10	82.37 (23.09)	78	93.55 (23.96)	97	−5.96 (−10.61 to −1.31)	.01	−0.26 (−0.47 to −0.06)
**Worry, PSWQ, total scores**							
Week 5	51.19 (17.39)	88	60.58 (15.65)	98	−7.47 (−11.14 to −3.80)	<.001	−0.52 (−0.78 to −0.27)
Week 10	49.51 (19.20)	78	58.86 (15.78)	97	−8.07 (−11.81 to −4.33)	<.001	−0.57 (−0.83 to −0.30)
**Perseverative thinking, PTQ, total scores**							
Week 5	32.58 (11.75)	88	36.88 (11.65)	98	−1.65 (−4.43 to 1.13)	.25	−0.17 (−0.47 to 0.12)
Week 10	30.36 (11.78)	78	36.88 (11.55)	97	−4.21 (−7.03 to −1.39)	.004	−0.45 (−0.74 to −0.15)
**Chronotype, MCTQ, midpoint of sleep, sleep corrected, h:min, min**							
Week 5	03:17 (61.82)	54	03:28 (83.41)	65	4.48 (−13.88 to 22.84)	.63	0.06 (−0.18 to 0.29)
Week 10	03:02 (61.79)	54	03:36 (79.63)	66	−15.22 (−34.13 to 3.70)	.12	0.12 (−0.43 to 0.05)
**Chronotype, MCTQ, Social jet lag, min**							
Week 5	25.67 (33.47)	72	34.69 (34.97)	80	−11.18 (−21.34 to −1.02)	.03	−0.30 (−0.58 to −0.03)
Week 10	27.34 (38.09)	69	34.65 (33.39)	80	−5.57 (−15.79 to 4.66)	.29	−0.15 (−0.43 to 0.13)
**Emotional categorization, ECAT, reaction time: positive words, ms**							
Week 5	1386.64 (175.27)	88	1412.45 (223.29)	98	−45.81 (−95.01 to 3.39)	.07	−0.21 (−0.44 to 0.02)
Week 10	1408.65 (215.84)	78	1388.09 (210.11)	96	−1.44 (−51.81 to 48.93)	.96	−0.01 (−0.24 to 0.23)
**Emotional categorization, ECAT, reaction time: negative words, ms**							
Week 5	1451.94 (222.06)	88	1479.79 (226.74)	98	−37.30 (−88.65 to 14.05)	.16	−0.17 (−0.41 to 0.06)
Week 10	1387.22 (177.29)	77	1412.58 (227.02)	96	−31.00 (−84.12 to 22.12)	.25	−0.14 (−0.39 to 0.10)
**Emotion recognition memory, EMEM, accuracy: positive words, %**							
Week 5	76.05 (13.15)	88	75.97 (13.45)	98	−1.01 (−4.34 to 2.32)	.56	−0.09 (−0.39 to 0.21)
Week 10	72.69 (11.96)	78	73.48 (11.12)	97	−1.95 (−5.38 to 1.48)	.26	−0.18 (−0.49 to 0.13)
**Emotion recognition memory, EMEM, accuracy: negative words, %**							
Week 5	75.26 (13.15)	88	74.64 (13.52)	98	0.19 (−3.20 to 3.58)	.91	0.02 (−0.28 to 0.31)
Week 10	70.29 (11.88)	78	69.51 (11.24)	97	−0.16 (−3.63 to 3.31)	.93	−0.01 (−0.32 to 0.29)

Abbreviations: CBT-I, Cognitive Behavioral Therapy for Insomnia; DERS, Difficulties in Emotional Regulation Scale; EMEM, Emotion Recognition Memory Task; FERT, Facial Expression Recognition Task; GCSE, General Certification for Secondary Education; ISI, Insomnia Severity Index; MCTQ, Munich Chronotype Questionnaire; PANAS, Positive and Negative Affect Schedule; PHQ-9, Patient Health Questionnaire-9; PSQI, Pittsburgh Sleep Quality Index; PSWQ, Penn State Worry Questionnaire; PTQ, Perseverative Thinking Questionnaire; SHE, Sleep Hygiene Education; SOL, sleep onset latency; TST, total sleep time.

^a^ Adjusted for baseline outcome measure, treatment assignment, assessment time, a treatment by time interaction and stratification variables (sex, age, baseline ISI and baseline PHQ-9).

^b^ Coprimary outcomes are presented as 97.5% CI, *P* < .025 considered significant for coprimary outcomes.

For the coprimary outcomes (FERT) at week 10, the estimated adjusted mean difference for accuracy of happy facial expressions was 3.01 (97.5% CI, −1.67 to 7.69; *P* = .15; Cohen *d* = 0.24) and −0.54 (97.5% CI, −3.92 to 2.84; *P* = .72; Cohen *d* = −0.05) for sad facial expressions, indicating no significant group differences (Table 2). Effects for both primary outcomes were consistent across sensitivity analyses investigating assumptions regarding outcome missingness (eTable 6 in Supplement 2), following multiple imputations of missing outcome data (eTable 6 in Supplement 2), when considering the effect of treatment adherence (eTable 9, eTable 10 in Supplement 2) and in a completely unadjusted model (eTable 6 in Supplement 2). In exploratory moderation analyses of the primary outcomes at 10 weeks, we found no significant subgroup differences by baseline levels of depression severity, worry, emotional regulation difficulties, or midpoint of sleep (eTable 12 in Supplement 2).

**Figure 2.**
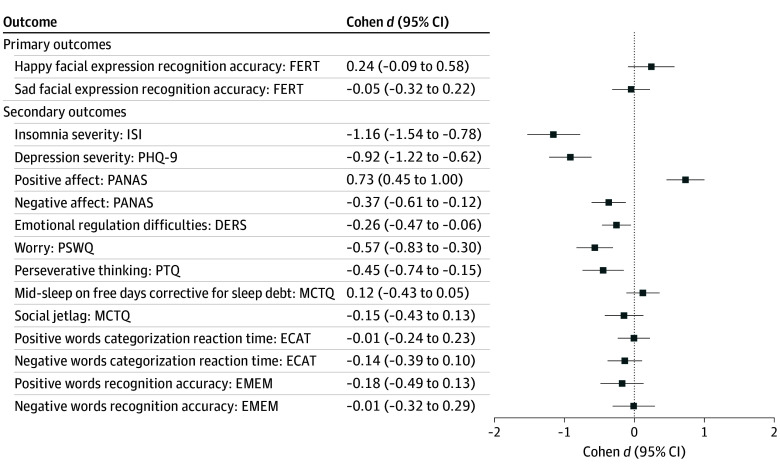
Standardized Effect Sizes (Cohen *d*) for Cognitive Behavioral Therapy for Insomnia CBT-I vs SHE on Outcomes at Week 10 CBT-I indicates cognitive behavioral therapy for insomnia; DERS, Difficulties in Emotion Regulation Scale; ECAT, emotion categorization task; EMEM, emotional recognition memory task; FERT, Facial Expression Recognition Task; ISI, Insomnia Severity Index; MCTQ, Munich Chronotype Questionnaire; PANAS, Positive and Negative Affect Schedule; PHQ-9, Patient Health Questionnaire-9; PSWQ, Penn State Worry Questionnaire; and PTQ, Perseverative Thinking Questionnaire.

At week 5, a small treatment effect was observed for recognition accuracy of happy facial expressions (4.53; 95% CI, 0.5-8.53; Cohen *d* = 0.37), with the CBT-I group performing better in identifying happy facial expressions. There was no significant group difference at 5 weeks for sad facial expressions (−0.50; 95% CI, −3.40 to 2.40; Cohen *d* = −0.05) (Table 2). Descriptive data for tertiary outcomes from the FERT (recognition accuracy of other facial expressions, misclassification scores, and reaction time for happy and sad facial expressions) can be found in eTable 11 in Supplement 2. Figure 2 shows the standardized effect sizes for all outcomes at week 10.

The CBT-I group reported significantly higher positive affect (PANAS) at both 5 weeks (1.88; 95% CI, 0.05-3.71; Cohen *d* = 0.27) and 10 weeks (4.99; 95% CI, 3.13-6.85; Cohen *d* = 0.73) and lower negative affect (PANAS) at 5 weeks (−3.63; 95% CI, −5.42 to −1.84; Cohen *d* = −0.48) and 10 weeks (−2.75; 95% CI, −4.58 to −0.92; Cohen *d* = −0.37). Treatment effects in favor of CBT-I were also observed for emotional regulation difficulties (DERS) at 5 weeks (−6.04; 95% CI, −10.61 to −1.47 Cohen *d* = −0.27) and 10 weeks (−5.96; 95% CI, −10.61 to −1.31; Cohen *d* = −0.26), and worry (PSWQ) at 5 weeks (−7.47; 95% CI, −11.14 to −3.80; Cohen *d* = −0.52) and 10 weeks (−8.07; 95% CI, −11.81; −4.33; Cohen *d* = −0.57). There was no significant group difference at week 5 for perseverative negative thinking (−1.65; 95% CI, −4.43 to 1.13; Cohen *d* = −0.17), but the CBT-I group reported lower scores at week 10 (−4.21; 95% CI, −7.03 to −1.39; Cohen *d* = −0.45). There were no significant group differences at 5 or 10 weeks for midpoint of sleep (week 5: 4.48; 95% CI, −13.88 to 22.84; Cohen *d* = 0.06; week 10: −15.22; 95% CI, −34.13 to 3.70; Cohen *d* = 0.12). Treatment effects in favor of CBT-I were observed for social jet lag at 5 weeks (−11.18; 95% CI, −21.34 to −1.02; Cohen *d* = −0.30), reflecting lower levels of social jet lag, but no significant group difference was observed at week 10 (−5.57; 95% CI, −15.79 to 4.66; Cohen *d* = −0.15).

There were no significant group differences for categorization performance (ECAT) or recognition memory of positive or negative words (EMEM) at 5 or 10 weeks (Cohen *d* range = −0.21 to 0.02) (Table 2). Descriptive data for tertiary outcomes on the ECAT task (accuracy and misses of positive and negative words) and EMEM task (misclassification and reaction time of positive and negative words) can be found in eTable 11 in Supplement 2.

### Mediation Analysis 

Four putative mediators showed between-group differences at both weeks 5 and 10 (emotional regulation difficulties, worry, positive affect, and negative affect) and were therefore assessed for mediation of the depression treatment effect. In mediation analyses, reduction in negative affect, emotional regulation difficulties, and worry at week 5 significantly mediated the treatment effect (range of mediated effect, 21.9% for Difficulties in Emotional Regulation Scale to 29.7% for Positive and Negative Affect Schedule) on the PHQ-9 at week 10 (Table 3; eFigure in Supplement 2). Use of adjunctive therapies for sleep or mental health was low and broadly comparable between trial arms during the study period (eTable 13 in Supplement 2).

**Table 3.** Mediation Analysis^a^

Mediators	Total effect		Direct effect		Indirect effect		Mediation, %
	Effect size (95% CI)	*P* value	Effect size (95% CI)	*P* value	Effect size (95% CI)	*P* value	
DERS (wk 5)	−3.51 (−4.81 to −2.16)	<.001	−2.74 (−4.05 to −1.51)	<.001	−0.77 (−1.46 to −0.13)	.01	21.9
PSWQ (wk 5)	−3.55 (−4.85 to −2.14)	<.001	−2.69 (−3.96 to −1.29)	<.001	−0.87 (−1.51 to −0.30)	.002	24.4
PANAS: PA (wk 5)	−3.53 (−4.86 to −2.20)	<.001	−3.09 (−4.31 to −1.89)	<.001	−0.44 (−1.12 to 0.16)	.15	12.4
PANAS: NA (wk 5)	−3.42 (−4.85 to −2.10)	<.001	−2.41 (−3.66 to −1.20)	<.001	−1.02 (−1.71 to −0.35)	<.001	29.7

Abbreviations: DERS, Difficulties in Emotional Regulation Scale; PANAS, Positive and Negative Affect Schedule; PSWQ, Penn State Worry Questionnaire.

^a^ Mediation analysis testing the effect of treatment allocation (cognitive behavioral therapy for insomnia CBT-I vs sleep hygiene education) on depression outcome (week 10) through putative mediators (week 5). Adjusted model (baseline measure of mediator + stratification variables: sex, age, baseline ISI, and baseline Patient Health Questionnaire-9).

### Safety Reporting 

No serious adverse events were reported to the trial team. Fewer participants in the CBT-I group reported suicidal ideation or mood instability compared with the SHE group at both 5 and 10 weeks (eTable 13 in Supplement 2).

## Discussion

We performed a randomized clinical trial to test whether emotion processing, reflected in perception of emotional facial expressions, was modified following CBT for insomnia. Treatment uptake was good and consistent with or higher than previous trials of automated digital CBT-I.^35,36,49,50^ The CBT-I group did not differ from control at 10 weeks for either of our coprimary outcomes (recognition accuracy of happy or sad facial expressions), yet we observed large clinical effects for insomnia and depression symptoms.

Our results suggest that improvement in depressive symptoms is not contingent on change in negative cognitive bias as reflected in the perception of emotional facial expressions. Baseline values for happy and sad facial expressions were similar to another large trial in depression^51^ and altered relative to controls.^52,53^ This suggests that our sample may exhibit biased perception at baseline, with scope for change following treatment, but it is important to note that the current study did not include a comparison group without depression. We limited confounding effects of concurrent treatment by excluding people who, at screening, were taking psychotropic medications or engaged in other treatments for sleep and mental health; and the number of participants reporting use of such therapies during the trial was low. CACE analysis showed that the treatment effect remained similar and nonsignificant when taking into account treatment adherence, and therefore our null results are unlikely to be explained by insufficient treatment engagement and/or response, especially given robust effects on insomnia and depression. Our findings contrast with small studies of antidepressants^16,17,18^ but are consistent with studies showing no concurrent or longitudinal association between recognition of happy or sad faces and depressive symptoms.^54,55^ It is possible that other types of tasks or the assessment of different cognitive-emotional processes may have shown sensitivity to sleep intervention, but this requires dedicated enquiry, and our emotional categorization and recognition memory tasks similarly revealed no group difference.

Improvement in depressive symptoms was explained, in part, by reductions in negative affect, emotional regulation difficulties, and worry. These partial mediators are conceptually and statistically related, and while significant, the magnitude of mediation was modest, ranging between 22% and 30% of the total effect. Our findings are consistent with a meta-analysis showing a medium effect size for reduction in worry following CBT-I.^56^ Worry is a maladaptive emotional regulation strategy that is associated with the generation and maintenance of negative mood states^57^ and depressive symptoms.^58,59,60^ Components and skills taught within the CBT-I program (eg, progressive muscle relaxation, mindfulness, cognitive restructuring) may defuse or limit the potential for worry, especially sleep-related worry. Directly improving sleep continuity, architecture, and regularity through behavioral therapy^61,62,63^ may improve functional connectivity between brain networks involved in the cognitive control of emotion and reduce negative affect,^64,65^ but this awaits empirical scrutiny. Other potential mechanisms and pathways also require consideration, including reduced sleepiness and fatigue, which may impact behavioral activation and social engagement and improve mood.

### Strengths and Limitations

Our study has several strengths. We used a task with previous sensitivity to the diagnosis and treatment of depression, we recruited a sample not engaged in concurrent treatments, and participants in the intervention arm engaged well with CBT-I, showing large improvements in insomnia and depressive symptoms.

The study also has limitations. While our sample size was sufficient to detect effects in the medium range, much smaller effects may not have been discernible. Small effect sizes in the expected direction were observed for improvement in the recognition of happy faces at 5 weeks (Cohen *d* = 0.37) and 10 weeks (Cohen *d* = 0.24). It may be that these effects represent realistic between-group change following intervention, but we believe it argues against a pivotal role in the reduction of depressive symptoms. The heterogeneity of depression and its varied underlying biotypes may render it difficult to detect reliable group-level changes in objective cognitive-emotional task performance.^66^ While our sample met PHQ-9 criteria for depressive symptom caseness, we did not confirm diagnosis via clinical interview. Nevertheless, the PHQ-9 has good sensitivity and specificity for major depressive disorder,^67,68^ and baseline questionnaire scores across various measures were consistent with published studies recruiting clinically defined populations.^69,70,71^ The sample was also predominately of White race, and for mechanistic reasons we excluded individuals who were taking psychotropic medications; this limits generalizability to many patients in clinical practice. Retention was good but there was greater outcome missingness in the CBT-I arm at week 10, which we attribute to the offer of CBT-I to the control arm following completion of the study. Sensitivity analyses accounting for missingness delivered the same conclusion.

## Conclusions

In this randomized clinical trial, we did not find evidence that CBT-I engenders change in the perception of facial expressions at post treatment despite large improvements in insomnia and depression. Early change in negative affect, emotional regulation difficulties, and worry were associated with lagged depression outcomes. From a clinical perspective, our study findings underline the importance of addressing insomnia in the treatment of depression. Future studies are needed to examine potential mechanisms of sleep intervention on depressive symptoms, incorporating assessments of sleep physiology, brain function, and high-frequency sampling of depressive symptoms and associated processes.

## Notes

Supplement 1.
Trial Protocol

Supplement 2. **eTable 1.** List of Reasons for Online Screening Exclusion **eTable 2.** Reasons for Withdrawal From the Study **eTable 3.** Odds Ratio of the Risk of Insomnia and Depression **eTable 4.** Missing Data on the Primary Outcome (FERT) **eTable 5.** Outcome Missingness on the Primary Outcome (FERT) at 10 Weeks **eTable 6.** Sensitivity Analyses of the Primary Outcome at 10 Weeks **eTable 7.** Total Number of Completed Sessions in Sleepio From the Treatment Group **eTable 8.** Baseline Characteristics of Complier (Completed ≥3 Sessions) vs Non-complier (Completed <3 Sessions) in the Treatment Group **eTable 9.** Primary Outcomes of Complier vs Non-Complier in the Treatment Group **eTable 10.** Complier Average Causal Effect (CACE) Analysis **eTable 11.** Means and Standard Deviation on Tertiary Outcomes Across Timepoints **eTable 12.** Subgroup Analysis of the Primary Outcomes (FERT) at 10 Weeks **eTable 13.** Descriptive Data on Suicidal Ideation, Mood Instability, and Treatment Engagement **eFigure.** Standardised Effects of CBT-I on Depressive Symptoms Week 10) in Mediation Models with Mediators (Week 5), Including Emotional Regulation Difficulties (A), Worry (B), Positive Affect (C), and Negative Affect (D) **eTable 14.** Sleepio Content per Session **eTable 15.** The Breakdown of Sleep Restriction Therapy Procedures (Session 3-5) Within Sleepio **eMethods.** Data Cleaning on MCTQ Variables

Supplement 3.
Data Sharing Statement
