# Supplementary material for: Emotional Processing Following Digital Cognitive Behavioral Therapy for Insomnia in People With Depressive Symptoms: A Randomized Clinical Trial
Source: JAMA Netw Open. 2025 Feb 27;8(2):e2461502. doi: 10.1001/jamanetworkopen.2024.61502 (PMC11868973; doi:10.1001/jamanetworkopen.2024.61502)
Supplement: Supplement 1. — Trial Protocol [file jamanetwopen-e2461502-s001.pdf]

The effect of digital cognitive behavioural therapy for insomnia on emotional processing in individuals with depressive symptoms (EPIC): A randomised controlled trial

Trial Protocol 2

Statistical Analysis Plan 18

Trial Protocol

Emotional **P**rocessing in **I**nsomnia **C**o-occurring with low mood (The EPIC study): study protocol

S. Tamm[1], K. Saunders[1], C. Harmer[2], S. Kyle[1.]

1: Department of Psychiatry, University of Oxford

2: Sleep and Circadian Neuroscience Institute (SCNi), Nuffield Department of Clinical Neurosciences, University of Oxford.

Background

Insomnia disorder is defined as persistent difficulties with sleep initiation and/or maintenance, resulting in significant daytime impairment. Insomnia affects approximately 10–12% of the adult population and is associated with increased risk for cardiovascular disease, depression, and early mortality (Morin et al., 2015; Morin & Benca, 2012). Maladaptive emotion regulation strategies, such as worry and rumination, are prominent features of both insomnia disorder and depression (Carney et al., 2010; Liu & Thompson, 2017). While these maladaptive strategies confer risk for depression (Wilkinson et al., 2013), recent data show that they also mediate the relationship between poor sleep quality and depressive symptoms in both longitudinal (O'Leary et al., 2017) and cross-sectional (Hom et al., 2016) analyses. Experimental laboratory studies have shown that sleep restriction in healthy participants results in decreased ability to down-regulate emotions in response to emotional stimuli (Mauss et al., 2013; Reddy et al., 2017; Tamm, Sandra et al., 2019) and sleep deprivation engenders impairments in emotion perception, attention and memory, as well as alterations in the neurophysiological structures sub-serving these functions (Krause et al., 2017). However, whether treatment of insomnia with cognitive behavioural therapy (CBT) leads to improved emotional processing is not known.

Given the strong rates of comorbidity between insomnia and depression (Staner, 2010) and the robust link between sleep and emotional processes, it is reasonable to suggest that persistent sleep disruption may confer risk for depression onset and maintenance via altered emotional states (Hertenstein et al., 2019). While mood disturbance features as a consequence of insomnia in diagnostic nosologies (American Psychological Association (APA), 2013; Medicine, 2001), there has been little evaluation of the impact of insomnia treatments on emotion regulation and perception. Trials of Cognitive Behavioural Therapy for Insomnia (CBT-I) in clinical and non-clinical samples show that insomnia treatment improves depressive

symptoms (Batterham et al., 2017; Blom et al., 2015; Christensen et al., 2016; Henry et al., 2020) however, there has been limited assessment of underlying mechanistic processes.

The present study is a refinement of a previously approved and performed study (The SLEEPER study, CUREC ethics approval reference R55815) in which the aim was to investigate the extent to which digital CBT-I (dCBT-I) modifies subjective and objective measures of emotional processing and to determine if these processes act as mediators in the pathway between sleep restoration and reduction in depressive symptoms. In the previous study, 341 participants with insomnia and clinically-significant depressive symptoms participated in a parallel-group, randomized controlled trial (RCT) of CBT-I versus Sleep Hygiene Education (SHE). Assessments included self-report questionnaires and computerised cognitive tasks. For unknown reasons the uptake of the intervention in the previous study was unexpectedly low (only 33% of participants in the intervention group accessed the intervention) and the outcome data provided by participants was not readily interpretable.

The aim of the present study is to test if dCBT-I modifies emotional processing, including both objective measures of affective bias and self-report measures of emotional regulation, worry, perseverative negative thinking, mood and chronotype in patients with insomnia and comorbid depressive symptoms. Furthermore, it aims to examine if changes in emotional bias and/or emotional regulation mediate the effect of CBT-I on depressive symptoms. The protocol is modified compared to our previous study (described below) to enhance treatment adherence through increased contact with the study team and to extend the emotional test battery to include tests of emotional perception, categorization and memory.

The **primary** hypothesis for the trial is:

1. dCBT-I will improve objective biases in negative emotional processing of faces relative to Sleep Hygiene Education (SHE) (5 and 10 weeks post randomisation)
    a. *Reduced recognition accuracy in response to sad facial expressions in the Facial Emotional Recognition Task (FERT)*
    b. *Increased recognition accuracy in response to happy facial expressions in the FERT*

The **secondary** hypotheses are:

1. dCBT-I will improve objective negative biases in categorization of emotional self-descriptors (5 and 10 weeks post randomisation) relative to SHE.
    1. *Decreased reaction time to identify positive compared with negative characteristics in Emotional Categorisation Task (ECAT)*
2. dCBT-I will increase positive emotional memory bias relative to SHE (10 weeks post randomisation)
    1. *Increased memory for positive compared with negative words in the Emotional Memory Task (EMEM)*
3. dCBT-I will reduce self-reported emotion regulation deficits (Difficulty with Emotion Regulation Scale, DERS), worry (Penn State Worry Questionnaire, PSWQ) and perseverative thinking (Perseverative Thinking Questionnaire, PTQ) (5 and 10 weeks post randomisation) relative to SHE.
4. dCBT-I will reduce self-reported depression severity (Patient Health Questionnaire, PHQ-9) (5 and 10 weeks post randomisation) relative to SHE.
5. dCBT-I will reduce insomnia severity (Insomnia Severity Index, ISI) (5 and 10 weeks post randomisation) relative to SHE.
6. Change in emotional perception of faces (FERT: *Changes in recognition accuracy for happy and sad facial expressions*) at week 5 will mediate change in depressive symptoms (PHQ-9) at week 10.

7. Change in emotion processing (ECAT: *reaction times for positive compared with negative words*) at week 5 will mediate change in depressive symptoms (PHQ-9) at week 10.
8. Change in self-rated emotion regulation, worry and preservative thinking (reductions in DERS, PSWQ and PTQ scores) at week 5 will mediate the effect of dCBT-i over SHE on depressive symptoms (PHQ-9) at week 10.
9. dCBT-I will increase the level of morningness (Munich ChronoType Questionnaire, MCTQ) (5 and 10 weeks post randomisation) relative to Sleep Hygiene Education (SHE).
    1. *Earlier midpoint of sleep on work-free days, sleep-corrected (MSF_{SC})*
10. dCBT-I will increase positive affect (Positive and Negative Affect Schedule Short Form - Positive affect, PANAS-SF: PA) and reduce negative affect (Positive and Negative Affect Schedule Short Form - Negative affect, PANAS-SF: NA) (5 and 10 weeks post randomisation) relative to SHE.
11. Change in level of morningness (MSF_{SC} from MCTQ) at week 5 will mediate change in depressive symptoms (Patient Health Questionnaire, PHQ-9) at week 10.
12. Change in mood (PANAS-SF: PA and NA) at week 5 will mediate change in depressive symptoms (Patient Health Questionnaire, PHQ-9) at week 10.

Methods/design

Research design

The study is a parallel-group, superiority RCT of dCBT-I versus SHE control. The study will be carried out online and through phone contact with the study team. Participants will provide informed consent online (*see **Appendix 1** for participant information sheet and **Appendix 2** for consent forms*) before carrying out screening online. Assessments (tasks and questionnaires), allocation to condition, and intervention will be carried out through web-based platforms. Online outcome assessment of our primary and secondary dependent variables will take place at 0 (baseline), 5 weeks post-randomisation (mid-treatment) and 10 weeks post-randomisation (post-treatment. At week 10 all participants allocated to the SHE will be offered dCBT-I to improve their insomnia (see Fig. 1 for trial design). Participants will be scheduled for a phone call before the baseline assessment to go through the study procedures, and 2 weeks post randomization to ensure that they have accessed the treatments.

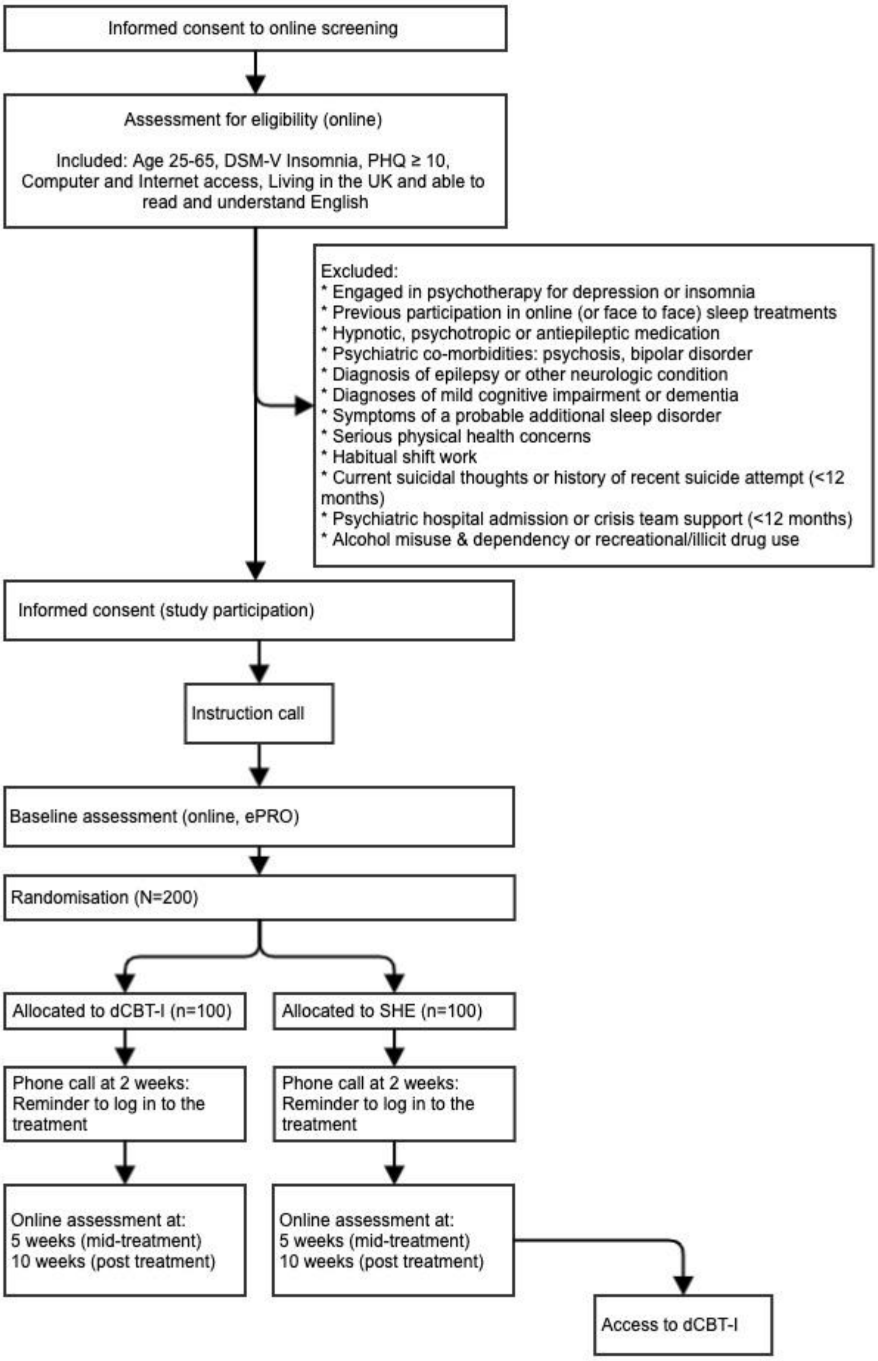

Figure 1: Flow chart diagram showing summary of the trial design for the **EPIC Study**. DSM-V Diagnostic and Statistical Manual of Mental Disorders, version 5, PHQ-9: Patient Health Questionnaire, dCBT-I digital Cognitive Behavioural Therapy for Insomnia, SHE Sleep Hygiene Education

Participants

We will recruit circa 200 community participants who report clinically-significant insomnia and depressive symptoms. Our inclusion criteria comprise of:

- A positive screen for probable DSM-5 insomnia disorder using items from the Sleep Condition Indicator (SCI) (scoring ≤2 on item 1 (sleep latency) or item 2 (wakefulness during the night) + scoring ≤2 on item 3 (frequency of disturbance) + scoring ≤1 on item 4 (sleep quality) + scoring ≤2 on daytime functioning items 5 or 6+scoring ≤2 on item 8 (chronicity of problem))
- Endorsement of depressive symptoms in the probable "caseness" range (PHQ score ≥10)
- Aged 25-65 years
- Access to a laptop or desktop computer and a phone and reliable internet access either at home or work
- Being able to read and understand English
- Currently living in the UK.

We will screen for comorbid conditions and medication use via an online survey and exclude people according to the following criteria:

- Engaged in psychotherapy for insomnia or depression
- Previous participation in online (or face to face) sleep treatments
- Hypnotic, psychotropic, or antiepileptic medications
- Psychiatric comorbidities: Psychosis or Bipolar Disorder
- Diagnosis of epilepsy or other neurological disorder
- Diagnoses of mild cognitive impairment or dementia.
- Symptoms of a probable additional sleep disorder (e.g. possible obstructive sleep apnea, restless legs syndrome)
- Serious physical health concerns necessitating surgery or with a survival prognosis of < 6 months
- Habitual night shift, evening, or rotating shift-workers (assessed during last month)
- Suicidal thoughts (Score >1 "yes" on C-SSRS suicide item 2), history of recent suicide attempt (<12 months)
- Psychiatric hospital admission in the past year, or crisis team support within the past year.
- Alcohol misuse & dependency or recreational/illicit drug use

Screenings

Screening will be performed online using the Qualtrics® platform, which will include a process of informed consent prior to any screening data being gathered. The screening questionnaire will automatically assess prospective participants according to the pre-determined inclusion and exclusion criteria (described above), and will include screening items for insomnia symptoms, (Sleep Condition Indicator (SCI) (C. A. Espie et al., 2014), depressive symptoms, (Patient Health Questionnaire (PHQ-9)) (Spitzer et al., 1999), Suicidal ideation and intent: Columbia-Suicide Severity Rating Scale (C-SSRS) (2 first items: Score >1 ("Yes") on item 2 = ineligible) probable Comorbid Sleep Disorders (Brief Sleep Disorders Screen) and alcohol misuse and dependency (score ≥ 8 on the Alcohol Use Disorders Identification Test (AUDIT)) (SAUNDERS et al., 1993). When a potential participant completes the online screening survey containing all items mentioned herein (**Appendix 3**) and provided informed consent (**Appendix 2**), an automatic email will be sent to the study coordinator. Ineligible participants will be notified automatically on completion of the online questionnaire. If ineligible participants are identified who are experiencing suicidal thoughts, they will automatically be provided with information that directs toward appropriate support (see section on assessment of safety). Eligible participants will be sent an email with a link to an electronic booking system (https://youcanbook.me/) where they can sign up for a phone call with the study coordinator. During the call, participants will be informed about the study procedure and have the possibility to ask any questions about the procedures. See **Appendix 4** for automatic emails, web page text and phone call script.

Interventions

dCBT-I

dCBT-I will be delivered using the Sleepio® programme (www.sleepio.com and associated Sleepio® app) (C. A. Espie et al., 2016; Freeman, Dunn, et al., 2015). The programme is fully automated and its underlying algorithms drive the delivery of information, support, and advice in a personally tailored manner. Delivery is structured into six weekly sessions, lasting an approximately 20 min each. The full programme can be accessed via the website or iOS app. Treatment content is based on CBT-I manuals and includes a behavioural component (sleep restriction, stimulus control, and relaxation), a cognitive component (paradoxical intention, cognitive restructuring, mindfulness, positive imagery, and putting the day to rest) and an educational component (psychoeducation and sleep hygiene) (C. A. Espie et al., 2001, 2007, 2008). The programme is highly interactive, and content is presented by an animated virtual therapist ('The Prof'). Before the start of the programme, participants complete a questionnaire to tailor the therapy and to set treatment goals. Participants complete daily sleep diary information throughout the intervention, which is used by the programme to provide tailored, personalised advice. Participants can opt to receive an email and/or SMS reminder each morning to prompt them to fill in their sleep diary. In addition, throughout the course of therapy, participants have access to an online community forum; weekly sleep expert sessions moderated by a clinical psychologist; and online library of information about sleep and sleep disorders. During weekly sleep expert sessions, users may vote on topics and submit questions to be addressed by the clinical psychologist. Questions are answered in such a way as to benefit as many people as possible and no personal medical advice is provided. Participants can view their online 'case file', which includes four sections: a progress review, a 'to-do' list, an agreed sleep schedule, and a list of further reading. The system provides online analytics which can be used to monitor adherence by assessing how many sessions were completed and the number of weeks to complete the course. All information gathered during the programme will be stored in encrypted form on secure servers. Automated dCBT-I using the Sleepio platform has been shown to be effective in reducing insomnia symptoms across >10 randomised controlled trials (Beattie et al., 2015; C. A. Espie et al., 2001, 2007, 2008, 2012, 2013; Freeman, Sheaves, et al., 2015; Henry et al., 2020). Further data from an uncontrolled study in a 'real-world' clinical setting supports the hypothesis that dCBT-I via Sleepio may improve depressive symptoms (Luik et al., 2017).

Sleep Hygiene Education (minimal treatment control)

Sleep hygiene education (SHE) will be provided to those in the control group as a minimal treatment control (Mohr et al., 2014), to control for some of the effects of engagement with a sleep intervention. SHE was selected on the basis that this it is the advice most frequently offered most typically in routine care (i.e general practice and primary care) (Everitt et al., 2014). To ensure consistency of approach and content, SHE will be delivered on a dedicated website where materials can be viewed and downloaded (hosted on Qualtrics.com). SHE will be based on recognised sleep hygiene advice (C. Espie, 2012) and will comprise behavioural advice concerning both lifestyle factors and environmental factors associated with sleep and sleeplessness. The latter, in particular, will focus on creating the optimal bedroom environment for good sleep. The content of SHE will cover the importance of limiting caffeine, nicotine, and alcohol and of carefully managing diet and exercise (lifestyle), as well as limiting noise and light, managing room temperature and body temperature, and improving air quality and bed comfort (environment). The study hypothesis surrounding sleep hygiene as a minimal treatment control will not be explicitly stated to participants, as the study will be presented as a comparison between two sleep interventions. On completion of the trial (week 10), all participants in the SHE arm will be offered access to dCBT-I.

Assessments

An electronic patient reported outcome (P1vital® ePRO) system, accessed via a study website, will be used to collect questionnaire and task performance data. The P1vital® ePRO system will issue email reminders to participants and study researchers when study-related activities are due. The P1vital® ePRO system will be setup, hosted and managed by P1vital Products Ltd and has been developed, validated and qualified in accordance with regulatory requirements for computerised systems used in clinical investigations. Participants will be advised to complete the online assessment in one sitting on a laptop or desktop computer. Prior to commencing an online assessment, participants are asked on what device they are using to complete the assessments (**Appendix 5a**).

Assessments will take place at weeks 0 (baseline), 5 (mid-treatment) and 10 weeks (post-treatment) (**see Fig. 2**). Participants are advised to allow enough time to complete the questionnaires (138 items in total) and emotional tasks in one session, on their home computer or laptop in a quiet, distraction free environment. Participants will also be advised to complete the tasks at the same time of day, where possible. Baseline variables include demographic information including: age, sex, ethnic group, employment status, highest education attained (**Appendix 6**). Frequency of sleep medication use and engagement with mental health services and treatments will be measured at weeks 5 and 10. Participants will be prompted by reminder emails to login and complete the online assessments according to fixed time-points. Participants will also receive a phone call after 2 weeks to ensure that they have accessed the treatment. For an itemised list of the outcome measures and procedures at each assessment see **Appendix 7**.

Primary Outcome measures

Our primary outcome measure will be the **Facial Expression Recognition Task** [FERT] (**Appendix 8**): The FERT assesses the interpretation of facial expressions (Harmer et al., 2009). Faces with six different basic emotions (happiness, fear, anger, disgust, sadness, surprise) are displayed on a screen and participants are required to indicate the expression on the face by selecting a corresponding button. The task takes approximately 15-20 minutes to complete and assesses the following dependent variables: Emotion Accuracy, Emotion Misclassifications and Emotion Reaction time.

Secondary Outcome measures (self-reported)

The Difficulties in Emotion Regulation Scale (**DERS, Appendix 5c)** is a self-report measure that assesses general deficits with emotion regulation. It has 36 items that are rated on a five-point Likert scale, ranging from 1 (almost never) to 5 (almost always). The scale is comprised of 6 factors: Non-acceptance of emotional responses (Non-Acceptance); difficulties engaging in goal-directed behaviour (Goal); impulse control difficulties (Impulse); lack of emotional awareness (Awareness); limited access to emotion regulation strategies (Strategy); lack of emotional clarity (Clarity). The DERS has high internal consistency (Cronbach's α = 0.93 for total DERS & Cronbach's α>:80 for each factors; test-retest = 0.87 for total DERS & ranging from 0.69 to 0.89 for all factors (Gratz 2004) and is sensitive to change following psychological therapy (Gratz 2006). In this study, the rubric of the DERS will be modified to refer to the "past week", in line with the other emotion regulation measures.

The Patient Health Questionnaire **PHQ9** (**Appendix 5b**) will be used to assess depressive symptoms in relation to each of the 9 DSM-IV criteria for depression as "0" (not at all) to "3" (nearly every day). Previous studies showed an effect size of 0.48 (Cohen's d) for dCBT-I compared to waitlist control/Sleep Hygiene Education after 8-10 weeks of treatment (Henry et al., 2020).

PSWQ-Past Week (Appendix 5d): The Penn State Worry Questionnaire is a commonly used measure of worry severity. The scale is a 16-item questionnaire that asks participants to rate 16 statements between 1 and 5. 1 being 'Not at all typical of me' and 5 being 'Very typical of me'. The 16-item PSWQ is a trait measure that assesses the generality of worry over time and situations, the intensity of the experience, and the uncontrollability of the process (Meyer 1990). The PSWQ-PW is an adaptation of the original PSWQ, containing 15 items that assesses state-dependent worry. The instructions ask participants to refer to "the past week" and the items are phrased in past-tense Stoeber 1998). The PSWQ-PW shows acceptable internal consistency Cronbach's alpha =.91 Stoeber 1998).

PTQ (Appendix 5g): The *Perseverative Thinking Questionnaire* (PTQ) (Ehring 2011), is a content-independent measure of repetitive negative thinking. The 15 item measure is comprised of three characteristic factors of repetitive negative thinking: (1a) repetitiveness (1b) intrusiveness, (1c) difficult to disengage from (2) unproductiveness (3) capturing mental capacity. The questionnaire has good internal consistency (Cronbach's α=.94; 15) and demonstrable sensitivity to change (Freeman 2015). 15 Items are rated on a 4-point Likert scale (range 0-60; with higher scores indicative of more perseverative thinking). In this study, the rubric of the PTQ will be modified to refer to the "past week" in line with the other emotion regulation measures.

Insomnia severity (Appendix 5e): Participants will complete the Insomnia Severity Index (ISI) (Morin 1993) to quantify global insomnia severity. The ISI is a seven-item insomnia assessment tool, probing both nighttime and daytime aspects of insomnia disorder, and is sensitive to change following CBT-I. It is a recommended outcome measure in insomnia trials (Buysse 2006). The ISI will be supplemented with four items from the Pittsburgh Sleep Quality Index (PSQI) (Buysse 1989) (**appendix 5g**) to permit calculation of quantitative sleep parameters, namely: total time in bed, sleep-onset latency, total sleep time, and sleep efficiency.

Mood Instability

Changes in mood instability will be assessed using one item (self-rated) from the Structured Clinical Interview for DSM-IV (SCID-II): *"Do you often have lots of sudden mood changes?".* In this study, the phrasing of the question is altered to refer to the past week. "*Over the past week, have you had lots of sudden mood changes?"*

Munich Chronotype Questionnaire (MCTQ, **Appendix 5j**) (Roenneberg et al., 2003) is a 14-item scale assessing the regularity of sleep timing on work days and work-free days. Midpoint of sleep on work-free days, sleep-corrected (MSF_{SC}) is calculated from the midpoint of sleep on work-free days minus half of the difference between sleep duration on work-free days and average sleep duration of the week to control for sleep debt.

Positive and Negative Affect Schedule – Short Form (PANAS-SF, Appendix 5k) (Watson et al., 1988)

is a 20-item scale measuring participants' positive and negative affect. Each item is a mood descriptor (e.g. interested). Participants are asked to rate their current mood using a 5-point scale (1 = very slightly or not at all; 5 = extremely). PANAS-SF consisted of two subscales: positive affect (PA) and negative affect (NA). Each subscale has 10-items. The PANAS-SF has been shown to have good internal consistency (Cronbach Alpha Coefficients: PA (past few weeks) = .87, NA (past few weeks) = .87).

Secondary Outcome measures (objective task performance)

Emotional Categorisation Task [ECAT]**,** The ECAT assesses speed to respond to positive and negative self-referent personality descriptors. Sixty personality characteristics selected to be disagreeable (e.g. "domineering", "untidy", "hostile") or agreeable (e.g. "cheerful", "honest", "optimistic") are presented. Participants are asked whether they would like or dislike to be referred to as each characteristic. The task takes approximately 5 minutes to complete and assess the following dependent variables: % Accuracy of each condition (positive or negative words), % misses of each condition (positive or negative words) and mean reaction time for correct responses for each condition (positive or negative words).

Emotion Recognition Memory Task [EMEM]**.** The EMEM measures recognition memory for affective words. Participants are presented with a series of words comprising the pleasant and unpleasant personality words that were previously presented to them in the ECAT, and a set of previously unseen distracter words. For each word, participants are required to report whether they have previously seen the word. The task takes approximately 10 minutes to complete and assesses the following dependent variables: % Accuracy of each condition (positive or negative words), misclassifications of each condition (positive or negative words), mean reaction time for correct responses for each condition (positive and negative words), target sensitivity for each condition and response bias for each condition.

Activity/Assessment	T-1	T0	T1	T2	T3
	Pre-Study	Baseline (Week 0)	2 weeks post randomisation	Mid-Treatment (Week 5)	Post-Treatment (Week 10)
Online Eligibility Screen (**Appendix 3**)	X				
Informed Consent	X				
Instruction call	X				
Randomisation		X (post baseline assessment)			
Interventions: DCBT-I and Sleep Hygiene Education		X (6 dCBT-I sessions completed over a minimum of 6 weeks)			
Demographics		X			
Follow-up call			X		
FERT **(Appendix 8)**		X		X	X
Self-report Emotion Regulation: DERS, PSWQ, PTQ (**Appendix 5c, 5d, 5g**)		X		X	X
Mood instability **(Appendix 5h)**		X		X	X
ISI (**Appendix 5e**)		X		X	X
PSQI (4 items) (**Appendix 5f**)		X		X	X
Depression (PHQ-9) (**Appendix 5b**)		X		X	X
Chronotype **(MCTQ, Appendix 5j**)		X		X	X

Positive and Negative Affect **(PANAS-SF, Appendix 5k**)		X		X	X
ECAT		X		X	X
EMEM		X		X	X
Audit of engagement with mental health services and sleep medication (**Appendix 5i**)				X	X
Device Monitoring Question(s) **(Appendix 5a)**		X		X	X

Fig 2: Standard Protocol Items: Recommendations for Interventional Trials (SPIRIT) figure.

Randomisation and allocation concealment

Randomization will be performed independently from the study team through the ePRO system. This study will use stratified randomisation with an allocation ratio of 1:1, as recommended for small scale clinical trials (Kernan et al., 1999). Randomisation and stratification according to sex (male/female), age (25-44, 45-65), depression severity (PHQ-9 Scores: ≤15 : ≥16) and insomnia severity (ISI scores ≤19 : ≥20). On completion of baseline measures each participant will be randomly assigned to either dCBT-I or SHE. The team will be unable to influence randomization in the ePRO system, but will manually contact the participant to inform them of their allocation.

Blinding and bias

Self-report assessments and performance tasks will be completed entirely online. Participants will be informed of their randomisation outcome (dCBT-I or SHE, referred to as sleep treatment 1 and 2 in the PIS) and will not be blind to treatment allocation. To minimise bias, the PIS will not explicitly reveal that SHE is used as a minimal control.

End of study

The end of study is defined as the last participants completed the last assessment (10 weeks).

Sample size calculation

Our planned primary intention-to-treat analysis will compare dCBT-I versus SHE for objective biases in negative emotional processing of faces at week 10. Our desired sample size of n=200 and an estimated attrition rate of 20% (based on recently completed trials (Kyle et al., 2017)), will be powered to detect a minimum between group standardized effect size of 0.5 at week 10, with a power of 90% and a two-tailed α of 0.05.

Recruitment and payment

The study will recruit through multiple channels. These may include online (e.g., Social media, mental health charity websites), print and broadcast media advertisements, and the use of contact lists where adults who have agreed to be contacted about future studies will be notified about the trial, (including Sleepio mailing lists). See **appendices 4, 9** and **10.** In addition to receiving free access to the dCBT-I program, participants will also receive payment in the form of Amazon™ gift vouchers for completing each post-randomisation assessment point (£10 for mid treatment, £15 for post-treatment).

Assessment of safety

The likelihood of serious adverse events occurring during this trial is low since CBT-I (in any format) has not been reported to cause them. The intervention offered in the trial has previously been tested in > 10 randomised trials and no SAEs related to the intervention have been reported (Beattie et al., 2015; C. A. Espie et al., 2001, 2007, 2008, 2012, 2013; Freeman, Sheaves, et al., 2015; Henry et al., 2020). Moreover, clinical evaluation in patients with anxiety disorder and depression revealed no evidence of SAEs (Gratz & Gunderson, 2006). Since the trial is completed online, with limited formal participant contact, we are unlikely to become aware of all potential adverse events. However, should we do so, we will define serious adverse events as: (1) death, (2) suicide attempt, and (3) admissions to secure units.

Given the relationship between improved sleep quality and reduced suicidal ideation (Trockel et al., 2015), we do not expect any increase in suicidal ideation amongst the intervention group. Conversely, it is likely that we may observe reductions in suicidal ideation in the intervention arm. Nonetheless, suicidal ideation will be assessed at each point in both arms using item 9 of the PHQ-9 and participants who demonstrate frequent suicidal ideation (score >1) will be prompted with an automatically triggered email, sign-posting to appropriate support (see **appendix 4, section 7** and **appendix 11**). While we screen out people with suicidal ideation on study entry (using criterion described in screening section: C-SSRS responses), we will not remove participants who report suicidal ideation during the course of the trial. Should a participant report significant emotional distress (including suicidal thinking) to the research team, an experienced psychiatrist will be available to support and advise, and where necessary speak directly to the participant.

Stop-go pilot phase

In light of the low treatment adherence in our previous study (The SLEEPER study, CUREC ethics approval reference R55815), we will perform a stop-go pilot phase in an initial group of 50 participants (n=25 in each arm). Data on treatment adherence and completion of assessments will be evaluated in these participants and the trial team will consider stopping the trial if treatment adherence, defined as logging in to at least one session on Sleepio, is lower than 66% in the CBT-I arm. Should this criterion be met the trial will continue to recruit the remaining 150 participants; the 50 pilot participants will be included in the final analyses.

Data management

Access to Data

Participant information will be kept strictly confidential. Electronic data will be de-identified with a code and such data will be kept on firewall and password-protected computers. An electronic linking file will enable identification by matching the study ID to the participant name; this file will be encrypted and stored

securely in a separate folder. This linking file will be destroyed at the end of the study. Responsible members of the University of Oxford may be given access to data for monitoring and/or audit of the study to ensure compliance with regulations.

Data Recording and Record Keeping

The chief investigator will be responsible for data management and archiving. Data quality will be maintained through adherence to the University of Oxford's Policy on the Management of Research Data and Records. We will seek to collect screening questionnaires electronically using the Qualtrics® Survey platform online. All other questionnaire and performance task data will be collected and securely stored online using the P1vital® ePRO system. These data will be encrypted before being transferred to the research group for analysis. P1Vital will store study data until deletion is requested by the chief investigator. This will take place after the study has terminated and all relevant data has been transferred to the research group. Participants email addresses and any other personal information will be deleted from the system at the end of the study. A data agreement will be put in place between P1vital® and the University of Oxford for the purposes of delivering this service. As part of this agreement P1Vital reserve the right to use the anonymised emotional processing task data (FERT, ECAT, EMEM) and basic demographic data such as gender & age (unattached to names or identifiable information) for internal, non-commercial research purposes. This will be made clear in the PIS.

Data collected from potential participants who do not go on to enrol in the study will be stored until the end of the study and then destroyed. Participants' personal information (e.g., email address, phone number) will be retained until either a summary of the study results has been sent to participants, or 12 months has passed since the end of study, whichever comes first.

Sleepio data

The Big Health team will supply Sleepio access codes. The study team will then provide each of the study participants with one access code for Sleepio®. The Sleepio® programme requires participants to register via this access code to use the system, inputting their personal data including identifiers. This is the usual procedure for all users of this programme. The Sleepio® programme has its own privacy policy that can be accessed here: https://www.Sleepio.com/privacy/.

The Big Health team will send the research team information on the number and date of completed Sleepio sessions for each participant (linked to their access code) as well as completed sleep diaries during the treatment. This information will be sent in an encrypted, password-protected spreadsheet and will not include any personally identifiable information. This will be made clear to participants in the PIS.

Data sharing

For increased reproducibility we aim to publish a fully anonymised data set in an open repository after the study has been completed and main results reported. We will make this clear in the PIS and ask participants to consent to this use of their research data.

Statistical analysis

In accordance with Consolidated Standards of Reporting Trials (CONSORT) guidelines, we will record and report all participant flow (Moher 2010) in addition to providing descriptive statistics of recruitment, dropout, and completeness of interventions. The main efficacy analysis will be conducted on an intention-to-treat basis including all participants. Baseline characteristics will be presented by randomization. We will then test the hypothesis for between-group change in the primary and secondary outcomes at 5 weeks and 10 weeks using analysis of covariance with baseline outcome measure and treatment assignment as fixed effects, and apply standard regression diagnostics. The analysis will use statistical techniques for handling missing outcome data under a missing-at random assumption. If the efficacy analysis shows significant between-group differences in the subjective and objective emotion processing measures at 5 and 10 weeks, modern causal inference methods will be to investigate the mediation hypotheses. Parametric regression models will be employed to test for the indirect effect of emotion regulation/processing at week 5 on depression severity (PHQ-9) at 10 weeks. Since all the measures are continuous, the indirect effects are calculated by multiplying relevant pathways and bootstrapping is used to produce valid standard errors for the indirect effects. All analyses will adjust for baseline measures of the mediators, outcomes and putative measured confounders. Mediation analyses are potentially biased by measurement error in mediators and hidden confounding between mediators and outcomes and we will investigate the sensitivity of the estimates to these problems.

Dissemination

We will seek to publish the results of this study in peer reviewed journals, irrespective of magnitude or direction of effect. Findings will also be presented at both national and international scientific meetings. The results will be made available online wherever possible, if permitted by journal policies.

References

American Psychological Association (APA). (2013). Diagnostic and Statistical Manual of Mental Disorders: Depressive Disorders. In *Diagnostic and Statistical Manual of Mental Disorders,*. American Psychiatric Publishing, Inc. https://doi.org/10.1176/appi.books.9780890425596.dsm04

Batterham, P. J., Christensen, H., Mackinnon, A. J., Gosling, J. A., Thorndike, F. P., Ritterband, L. M., Glozier, N., & Griffiths, K. M. (2017). Trajectories of change and long-term outcomes in a randomised controlled trial of internet-based insomnia treatment to prevent depression. *BJPsych Open*, *3*(5), 228–235. https://doi.org/10.1192/bjpo.bp.117.005231

Beattie, L., Kyle, S. D., Espie, C. A., & Biello, S. M. (2015). Social interactions, emotion and sleep: A systematic review and research agenda. *Sleep Medicine Reviews*, *24*, 83–100. https://doi.org/10.1016/j.smrv.2014.12.005

Blom, K., Jernelöv, S., Kraepelien, M., Bergdahl, M. O., Jungmarker, K., Ankartjärn, L., Lindefors, N., & Kaldo, V. (2015). Internet Treatment Addressing either Insomnia or Depression, for Patients with both Diagnoses: A Randomized Trial. *Sleep*, *38*(2), 267–277. https://doi.org/10.5665/sleep.4412

Carney, C. E., Harris, A. L., Moss, T. G., & Edinger, J. D. (2010). Distinguishing rumination from worry in clinical insomnia. *Behaviour Research and Therapy*, *48*(6), 540–546. https://doi.org/10.1016/j.brat.2010.03.004

Christensen, H., Batterham, P. J., Gosling, J. A., Ritterband, L. M., Griffiths, K. M., Thorndike, F. P., Glozier, N., O’Dea, B., Hickie, I. B., & Mackinnon, A. J. (2016). Effectiveness of an online insomnia program (SHUTi) for prevention of depressive episodes (the GoodNight Study): a randomised controlled trial. *The Lancet. Psychiatry*, *3*(4), 333–341. https://doi.org/10.1016/S2215-0366(15)00536-2

Espie, C. (2012). *Overcoming insomnia and sleep problems: A self-help guide using cognitive behavioural techniques*. Robinson.

Espie, C. A., Fleming, L., Cassidy, J., Samuel, L., Taylor, L. M., White, C. A., Douglas, N. J., Engleman, H. M., Kelly, H. L., & Paul, J. (2008). Randomized controlled clinical effectiveness trial of cognitive behavior therapy compared with treatment as usual for persistent insomnia in patients with cancer. *Journal of Clinical Oncology*, *26*(28), 4651–4658. https://doi.org/10.1200/JCO.2007.13.9006

Espie, C. A., Hames, P., & McKinstry, B. (2013). Use of the internet and mobile media for delivery of cognitive behavioral insomnia therapy. In *Sleep Medicine Clinics* (Vol. 8, Issue 3, pp. 407–419). Elsevier. https://doi.org/10.1016/j.jsmc.2013.06.001

Espie, C. A., Inglis, S. J., Tessier, S., & Harvey, L. (2001). The clinical effectiveness of cognitive behaviour therapy for chronic insomnia: Implementation and evaluation of a sleep clinic in general medical practice. *Behaviour Research and Therapy*, *39*(1), 45–60. https://doi.org/10.1016/S0005-7967(99)00157-6

Espie, C. A., Kyle, S. D., Hames, P., Gardani, M., Fleming, L., & Cape, J. (2014). The Sleep Condition Indicator: A clinical screening tool to evaluate insomnia disorder. *BMJ Open*, *4*(3), e004183. https://doi.org/10.1136/bmjopen-2013-004183

Espie, C. A., Kyle, S. D., Williams, C., Ong, J. C., Douglas, N. J., Hames, P., & Brown, J. S. L. (2012). A randomized, placebo-controlled trial of online cognitive behavioral therapy for chronic insomnia disorder delivered via an automated media-rich web application. *Sleep*, *35*(6), 769–781. https://doi.org/10.5665/sleep.1872

Espie, C. A., Luik, A. I., Cape, J., Drake, C. L., Siriwardena, A. N., Ong, J. C., Gordon, C., Bostock, S., Hames, P., Nisbet, M., Sheaves, B., Foster, R., Freeman, D., Costa-Font, J., Emsley, R., & Kyle, S. D. (2016). Digital Cognitive Behavioural Therapy for Insomnia versus sleep hygiene education: The impact of improved sleep on functional health, quality of life and psychological well-being. Study protocol for a randomised controlled trial. *Trials*, *17*(1). https://doi.org/10.1186/s13063-016-1364-7

Espie, C. A., MacMahon, K. M. A., Kelly, H. L., Broomfield, N. M., Douglas, N. J., Engleman, H. M., McKinstry, B., Morin, C. M., Walker, A., & Wilson, P. (2007). Randomized clinical effectiveness trial of nurse-administered small-group cognitive behavior therapy for persistent insomnia in general practice. *Sleep*, *30*(5), 574–584. https://doi.org/10.1093/sleep/30.5.574

Everitt, H., McDermott, L., Leydon, G., Yules, H., Baldwin, D., & Little, P. (2014). GPs' management strategies for patients with insomnia: a survey and qualitative interview study. *The British Journal of General Practice : The Journal of the Royal College of General Practitioners*, *64*(619), e112-9. https://doi.org/10.3399/bjgp14X677176

Freeman, D., Dunn, G., Startup, H., Pugh, K., Cordwell, J., Mander, H., Černis, E., Wingham, G., Shirvell, K., & Kingdon, D. (2015). Effects of cognitive behaviour therapy for worry on persecutory delusions in patients with psychosis (WIT): A parallel, single-blind, randomised controlled trial with a mediation analysis. *The Lancet Psychiatry*, *2*(4), 305–313. https://doi.org/10.1016/S2215-0366(15)00039-5

Freeman, D., Sheaves, B., Goodwin, G. M., Yu, L. M., Harrison, P. J., Emsley, R., Bostock, S., Foster, R. G., Wadekar, V., Hinds, C., & Espie, C. A. (2015). Effects of cognitive behavioural therapy for insomnia on the mental health of university students: Study protocol for a randomized controlled trial. *Trials*, *16*(1). https://doi.org/10.1186/s13063-015-0756-4

Gratz, K. L., & Gunderson, J. G. (2006). Preliminary Data on an Acceptance-Based Emotion Regulation Group Intervention for Deliberate Self-Harm Among Women With Borderline Personality Disorder.

Behavior Therapy, *37*(1), 25–35. https://doi.org/10.1016/j.beth.2005.03.002

Harmer, C. J., O'Sullivan, U., Favaron, E., Massey-Chase, R., Ayres, R., Reinecke, A., Goodwin, G. M., & Cowen, P. J. (2009). Effect of acute antidepressant administration on negative affective bias in depressed patients. *American Journal of Psychiatry*, *166*(10), 1178–1184. https://doi.org/10.1176/appi.ajp.2009.09020149

Henry, A. L., Miller, C. B., Emsley, R., Sheaves, B., Freeman, D., Luik, A. I., Littlewood, D. L., Saunders, K. E. A., Kanady, J. C., Carl, J. R., Davis, M. L., Kyle, S. D., & Espie, C. A. (2020). Insomnia as a mediating therapeutic target for depressive symptoms: A sub-analysis of participant data from two large randomized controlled trials of a digital sleep intervention. *Journal of Sleep Research*. https://doi.org/10.1111/jsr.13140

Hertenstein, E., Feige, B., Gmeiner, T., Kienzler, C., Spiegelhalder, K., Johann, A., Jansson-Fröjmark, M., Palagini, L., Rücker, G., Riemann, D., & Baglioni, C. (2019). Insomnia as a predictor of mental disorders: A systematic review and meta-analysis. *Sleep Medicine Reviews*, *43*, 96–105. https://doi.org/10.1016/j.smrv.2018.10.006

Hom, M. A., Stanley, I. H., Rogers, M. L., Tzoneva, M., Bernert, R. A., & Joiner, T. E. (2016). The association between sleep disturbances and depression among firefighters: emotion dysregulation as an explanatory factor. *Journal of Clinical Sleep Medicine*, *12*(2), 235–245. https://doi.org/10.5664/jcsm.5492

Kernan, W. N., Viscoli, C. M., Makuch, R. W., Brass, L. M., & Horwitz, R. I. (1999). Stratified randomization for clinical trials. *Journal of Clinical Epidemiology*, *52*(1), 19–26. https://doi.org/10.1016/S0895-4356(98)00138-3

Krause, A. J., Simon, E. Ben, Mander, B. A., Greer, S. M., Saletin, J. M., Goldstein-Piekarski, A. N., & Walker, M. P. (2017). The sleep-deprived human brain. *Nature Reviews Neuroscience*, *18*(7), 404–418. https://doi.org/10.1038/nrn.2017.55

Kyle, S. D., Hurry, M. E. D., Emsley, R., Luik, A. I., Omlin, X., Spiegelhalder, K., Espie, C. A., & Sexton, C. E. (2017). Effects of digital Cognitive Behavioural Therapy for Insomnia on cognitive function: Study protocol for a randomised controlled trial. *Trials*, *18*(1), 1–8. https://doi.org/10.1186/s13063-017-2012-6

Liu, D. Y., & Thompson, R. J. (2017). Selection and implementation of emotion regulation strategies in major depressive disorder: An integrative review. *Clinical Psychology Review*, *57*, 183–194. https://doi.org/10.1016/j.cpr.2017.07.004

Luik, A. I., Bostock, S., Chisnall, L., Kyle, S. D., Lidbetter, N., Baldwin, N., & Espie, C. A. (2017). Treating Depression and Anxiety with Digital Cognitive Behavioural Therapy for Insomnia: A Real World NHS Evaluation Using Standardized Outcome Measures. *Behavioural and Cognitive Psychotherapy*, *45*(1), 91–96. https://doi.org/10.1017/S1352465816000369

Mauss, I. B., Troy, A. S., & LeBourgeois, M. K. (2013). Poorer sleep quality is associated with lower emotion-regulation ability in a laboratory paradigm. *Cogn Emot*, *27*(3), 567–576. https://doi.org/10.1080/02699931.2012.727783

Medicine, A. A. of S. (2001). The International Classification of Sleep Disorders: Diagnostic and Coding Manual. In *American Academy of Sleep Medicine*. https://doi.org/10.1212/wnl.41.1.160

Mohr, D. C., Ho, J., Hart, T. L., Baron, K. G., Berendsen, M., Beckner, V., Cai, X., Cuijpers, P., Spring, B., Kinsinger, S. W., Schroder, K. E., & Duffecy, J. (2014). Control condition design and implementation

features in controlled trials: a meta-analysis of trials evaluating psychotherapy for depression. In *Translational Behavioral Medicine* (Vol. 4, Issue 4, pp. 407–423). Springer New York LLC. https://doi.org/10.1007/s13142-014-0262-3

Morin, C. M., & Benca, R. (2012). Chronic insomnia. *The Lancet*, *379*(9821), 1129–1141. https://doi.org/10.1016/S0140-6736(11)60750-2

Morin, C. M., Drake, C. L., Harvey, A. G., Krystal, A. D., Manber, R., Riemann, D., & Spiegelhalder, K. (2015). Insomnia disorder. *Nature Reviews Disease Primers*, *1*, 15026. https://doi.org/10.1038/nrdp.2015.26

O'Leary, K., Bylsma, L. M., & Rottenberg, J. (2017). Why might poor sleep quality lead to depression? A role for emotion regulation. *Cognition and Emotion*, *31*(8), 1698–1706. https://doi.org/10.1080/02699931.2016.1247035

Reddy, R., Palmer, C. A., Jackson, C., Farris, S. G., & Alfano, C. A. (2017). Impact of sleep restriction versus idealized sleep on emotional experience, reactivity and regulation in healthy adolescents. *Journal of Sleep Research*, *26*(4), 516–525. https://doi.org/10.1111/jsr.12484

Roenneberg, T., Wirz-Justice, A., & Merrow, M. (2003). Life between clocks: Daily temporal patterns of human chronotypes. *Journal of Biological Rhythms*, *18*(1), 80–90. https://doi.org/10.1177/0748730402239679

SAUNDERS, J. B., AASLAND, O. G., BABOR, T. F., DE LA FUENTE, J. R., & GRANT, M. (1993). Development of the Alcohol Use Disorders Identification Test (AUDIT): WHO Collaborative Project on Early Detection of Persons with Harmful Alcohol Consumption-II. *Addiction*, *88*(6), 791–804. https://doi.org/10.1111/j.1360-0443.1993.tb02093.x

Spitzer, R. L., Kroenke, K., & Williams, J. B. W. (1999). Validation and utility of a self-report version of PRIME-MD: The PHQ Primary Care Study. *Journal of the American Medical Association*, *282*(18), 1737–1744. https://doi.org/10.1001/jama.282.18.1737

Staner, L. (2010). Comorbidity of insomnia and depression. *Sleep Medicine Reviews*, *14*(1), 35–46. https://doi.org/10.1016/j.smrv.2009.09.003

Tamm, Sandra, Nilsonne, G., Schwarz, J., Golkar, A., Kecklund, G., Petrovic, P., Fischer, H., Akerstedt, T., & Lekander, M. (2019). Sleep restriction caused impaired emotional regulation without detectable brain activation changes-A functional magnetic resonance imaging study. *Royal Society Open Science*, *6*(3). https://doi.org/10.1098/rsos.181704

Trockel, M., Karlin, B. E., Taylor, C. B., Brown, G. K., & Manber, R. (2015). Effects of cognitive behavioral therapy for insomnia on suicidal ideation in veterans. *Sleep*, *38*(2), 259–265. https://doi.org/10.5665/sleep.4410

Watson, D., Clark, L. A., & Tellegen, A. (1988). Development and validation of brief measures of positive and negative affect: the PANAS scales. *Journal of Personality and Social Psychology*, *54*(6), 1063–1070. https://doi.org/10.1037//0022-3514.54.6.1063

Wilkinson, P. O., Croudace, T. J., & Goodyer, I. M. (2013). Rumination, anxiety, depressive symptoms and subsequent depression in adolescents at risk for psychopathology: A longitudinal cohort study. *BMC Psychiatry*, *13*. https://doi.org/10.1186/1471-244X-13-250

Statistical Analysis Plan

Title: Emotional **P**rocessing in **I**nsomnia **C**o-occurring with low mood

Short title: The EPIC study

Ethical approval reference: R72715/RE003

Version number and date: 1.0 2022-04-14

This statistical plan supports the study protocol, version 1.2 (2021-03-26)

Written by: Sandra Tamm

Reviewed by: Jennifer Hellier (statistician)

Approved by: Simon D. Kyle (Chief Investigator)

Table of Contents

Background 20

Aim and hypotheses 20

Method 21

Study design 21
Inclusion/exclusion 22
Measurements 23
Randomisation and allocation concealment 24
Sample size calculation 24
Adverse events 24

Analysis – general considerations 24

Statistical software 24
Descriptive statistics 25
Characteristics of participants 25
Definition of population for analysis 26

Primary analysis 26

Primary outcome 26
Handling missing data 27
Handling outliers 27
Multiple comparisons and multiplicity 27
Model assumptions 27

Secondary analysis 27

Secondary outcomes 27
Treatment engagement 28
Mediation analysis 29
CACE analysis 29

Exploratory analysis 30

Sensitivity analysis 30

References 30

Background

Insomnia disorder is defined as persistent difficulties with sleep initiation and/or maintenance, resulting in significant daytime impairment. Insomnia affects approximately 10–12% of the adult population and is associated with increased risk for cardiovascular disease, depression, and early mortality (Morin et al., 2015; Morin and Benca, 2012). Maladaptive emotion regulation strategies, such as worry and rumination, are prominent features of both insomnia disorder and depression (Carney et al., 2010; Liu and Thompson, 2017). While these maladaptive strategies confer risk for depression (Wilkinson et al., 2013), recent data show that they also mediate the relationship between poor sleep quality and depressive symptoms in both longitudinal (O'Leary et al., 2017) and cross-sectional (Hom et al., 2016) analyses. Experimental laboratory studies have shown that sleep restriction in healthy participants results in decreased ability to down-regulate emotions in response to emotional stimuli (Mauss et al., 2013; Reddy et al., 2017; Tamm, Sandra et al., 2019) and sleep deprivation engenders impairments in emotion perception, attention and memory, as well as alterations in the neurophysiological structures sub-serving these functions (Krause et al., 2017). However, whether treatment of insomnia with cognitive behavioural therapy (CBT) leads to improved emotional processing is not known.

Aim and hypotheses

The aim of the present study is to test if digital cognitive behaviour therapy for insomnia (dCBT-I) modifies emotional processing, including both objective measures of affective bias and self-report measures of emotional regulation, worry, perseverative negative thinking, mood and chronotype in patients with insomnia and comorbid depressive symptoms. Furthermore, it aims to examine if changes in emotional bias and/or emotional regulation mediate the effect of CBT-I on depressive symptoms.

The **primary hypothesis** for the trial is:

1. dCBT-I will improve objective biases in negative emotional processing of faces relative to Sleep Hygiene Education (SHE) (10 weeks post randomisation)

    a. Reduced recognition accuracy in response to sad facial expressions in the Facial Emotional Recognition Task (FERT)

    b. Increased recognition accuracy in response to happy facial expressions in the FERT

The **secondary hypotheses** are:

1. dCBT-I will improve objective biases in negative emotional processing of faces relative to Sleep Hygiene Education (SHE) (5 weeks post randomisation)

2. dCBT-I will improve objective negative biases in categorization of emotional self-descriptors (5 and 10 weeks post randomisation) relative to SHE

    a. Decreased reaction time to identify positive compared with negative characteristics in Emotional Categorisation Task (ECAT)

3. dCBT-I will increase positive emotional memory bias relative to SHE (5 and 10 weeks post randomisation)

   a. Increased memory for positive compared with negative words in the Emotional Memory Task (EMEM)

4. dCBT-I will reduce self-reported emotion regulation deficits (Difficulty with Emotion Regulation Scale, DERS), worry (Penn State Worry Questionnaire, PSWQ) and perseverative thinking (Perseverative Thinking Questionnaire, PTQ) (5 and 10 weeks post randomisation) relative to SHE

5. dCBT-I will reduce self-reported depression severity (Patient Health Questionnaire, PHQ-9) (5 and 10 weeks post randomisation) relative to SHE

6. dCBT-I will reduce insomnia severity (Insomnia Severity Index, ISI) (5 and 10 weeks post randomisation) relative to SHE

7. Change in emotional perception of faces (FERT: Changes in recognition accuracy for happy and sad facial expressions) at week 5 will mediate change in depressive symptoms (PHQ-9) at week 10

8. Change in emotion processing (ECAT: reaction times for positive compared with negative words) at week 5 will mediate change in depressive symptoms (PHQ-9) at week 10.

9. Change in self-rated emotion regulation, worry and preservative thinking (reductions in DERS, PSWQ and PTQ scores) at week 5 will mediate the effect of dCBT-I over SHE on depressive symptoms (PHQ-9) at week 10

11. dCBT-I will increase the level of morningness (Munich ChronoType Questionnaire, MCTQ) (5 and 10 weeks post randomisation) relative to SHE

   a. Earlier midpoint of sleep on work-free days, sleep-corrected (MSF SC)

10. dCBT-I will increase positive affect (Positive and Negative Affect Schedule Short Form - Positive affect, PANAS-SF: PA) and reduce negative affect (Positive and Negative Affect Schedule Short Form - Negative affect, PANAS-SF: NA) (5 and 10 weeks post randomisation) relative to SHE

12. Change in level of morningness (MSF SC from MCTQ) at week 5 will mediate change in depressive symptoms (Patient Health Questionnaire, PHQ-9) at week 10

13. Change in mood (PANAS-SF: PA and NA) at week 5 will mediate change in depressive symptoms (PHQ-9) at week 10

Method

Study design

The study is a parallel-group, superiority RCT of digital Cognitive Behavioural Therapy for Insomnia (dCBT-I) versus Sleep Hygiene Education (SHE) control. The study is carried out online and through phone contact with the study team. Participants are screened and provide informed consent online. Assessments (tasks and questionnaires), allocation to condition, and intervention is carried out through web-based platforms. Online outcome assessment of our primary and secondary dependent variables take place at 0 (baseline), 5 weeks post-randomisation (mid-treatment) and 10 weeks post-randomisation (post-treatment). At week 10 all participants allocated to the SHE are offered dCBT-I to improve their insomnia. Participants are scheduled for a phone call before the baseline assessment to go

through the study procedures, and 2 weeks post randomization to ensure that they have accessed the treatments.

Inclusion/exclusion

We will recruit circa 200 community participants who report clinically-significant insomnia and depressive symptoms. Our inclusion criteria comprise of:

- A positive screen for probable DSM-5 insomnia disorder using items from the Sleep Condition Indicator (SCI) (scoring ≤2 on item 1 (sleep latency) or item 2 (wakefulness during the night) + scoring ≤2 on item 3 (frequency of disturbance) + scoring ≤1 on item 4 (sleep quality) + scoring ≤2 on daytime functioning items 5 or 6+scoring ≤2 on item 8 (chronicity of problem))
- Endorsement of depressive symptoms in the probable “caseness” range (PHQ score ≥10)
- Aged 25-65 years
- Access to a laptop or desktop computer and a phone and reliable internet access either at home or work
- Being able to read and understand English
- Currently living in the UK.

We will screen for comorbid conditions and medication use via an online survey and exclude people according to the following criteria:

- Engaged in psychotherapy for insomnia or depression
- Previous participation in online (or face to face) sleep treatments
- Hypnotic, psychotropic, or antiepileptic medications
- Psychiatric comorbidities: Psychosis or Bipolar Disorder
- Diagnosis of epilepsy or other neurological disorder
- Diagnoses of mild cognitive impairment or dementia.
- Symptoms of a probable additional sleep disorder (e.g. possible obstructive sleep apnea, restless legs syndrome)
- Serious physical health concerns necessitating surgery or with a survival prognosis of < 6 months
- Habitual night shift, evening, or rotating shift-workers (assessed during last month)
- Suicidal thoughts (Score >1 “yes” on C-SSRS suicide item 2), history of recent suicide attempt (<12 months)
- Psychiatric hospital admission in the past year, or crisis team support within the past year.
- Alcohol misuse & dependency or recreational/illicit drug use

Measurements

The primary and secondary outcome measures are collected at baseline, at 5 weeks and at 10 weeks. The 5 weeks assessment expires after 5 weeks (i.e. at the time of the last assessment) and the 10 weeks assessment expires 4 weeks after the scheduled date, and after the assessments expire participants are no longer able to complete the assessment. The following table lists the variables collected during the trial:

Type of variable	Measure	Pre-Study	Baseline (Week 0)	Mid-Treatment (Week 5)	Post-Treatment (Week 10)
	Online Eligibility Screen	X			
	Demographics		X		
Primary outcome and mediator	FERT		X	X	X
Secondary outcome and mediator	Self-report Emotion Regulation: DERS, PSWQ, PTQ		X	X	X
Secondary outcome	Mood instability		X	X	X
Secondary outcome	ISI		X	X	X
Secondary outcome	PSQI (4 items)		X	X	X
Secondary outcome	Depression (PHQ-9)		X	X	X
Secondary outcome and mediator	Chronotype		X	X	X
Secondary Outcome and mediator	Positive and Negative Affect		X	X	X
Secondary outcome and mediator	ECAT		X	X	X
Secondary outcome and mediator	EMEM		X	X	X
Auditory variable	Audit of engagement with mental health services and sleep medication			X	X
Auditory variable	Device Monitoring Question(s)		X	X	X

Randomisation and allocation concealment

Randomization will be performed independently from the study team through the ePRO system. This study will use stratified randomisation with an allocation ratio of 1:1. Randomisation and stratification according to sex (male/female), age (25-44, 45-65), depression severity (PHQ-9 Scores: ≤15 : ≥16) and insomnia severity (ISI scores ≤19 : ≥20). On completion of baseline measures each participant will be randomly assigned to either dCBT-I or SHE. The team will be unable to influence randomization in the ePRO system, but will manually contact the participant to inform them of their allocation.

Sample size calculation

Our planned primary intention-to-treat analysis will compare dCBT-I versus SHE for objective biases in negative emotional processing of faces at week 10. Our desired sample size of n=200 and an estimated attrition rate of 15% (based on recently completed trials (Kyle et al., 2017)), was powered to detect a minimum between group standardized effect size of 0.5 at week 10, with a power of 90% and a two-tailed α of 0.05.

Considering the co-primary outcome, alpha will be adjusted to 0.025 to control the overall type 1 error rate. Therefore, conservatively assuming an alpha of 0.025 and no correlation between the outcomes; the study would be able to detect an effect size of 0.5 with 85% power on the two primary outcomes (recognition accuracy in response to happy and sad facial expressions). The sample size remains inflated to allow for a rate of by 15% lost to follow up.

Adverse events

Since the trial is completed online, with limited formal participant contact, we are unlikely to become aware of all potential adverse events. However, should we do so, we will define serious adverse events as: (1) death, (2) suicide attempt, and (3) admissions to secure units.

Analysis – general considerations

Statistical software

Analyses and processing of the data will be performed in R (R Core Team (2020). R: A language and environment for statistical computing. R Foundation for Statistical Computing, Vienna, Austria. https://www.R-project.org/). Mixed effect models will be run using the lme4 and nlme packages, CACE analyses will be performed using the eefAnalytics package and mediation analyses will be performed using the MEDIATION package.

Descriptive statistics

We will present the data using a CONSORT diagram, see figure below.

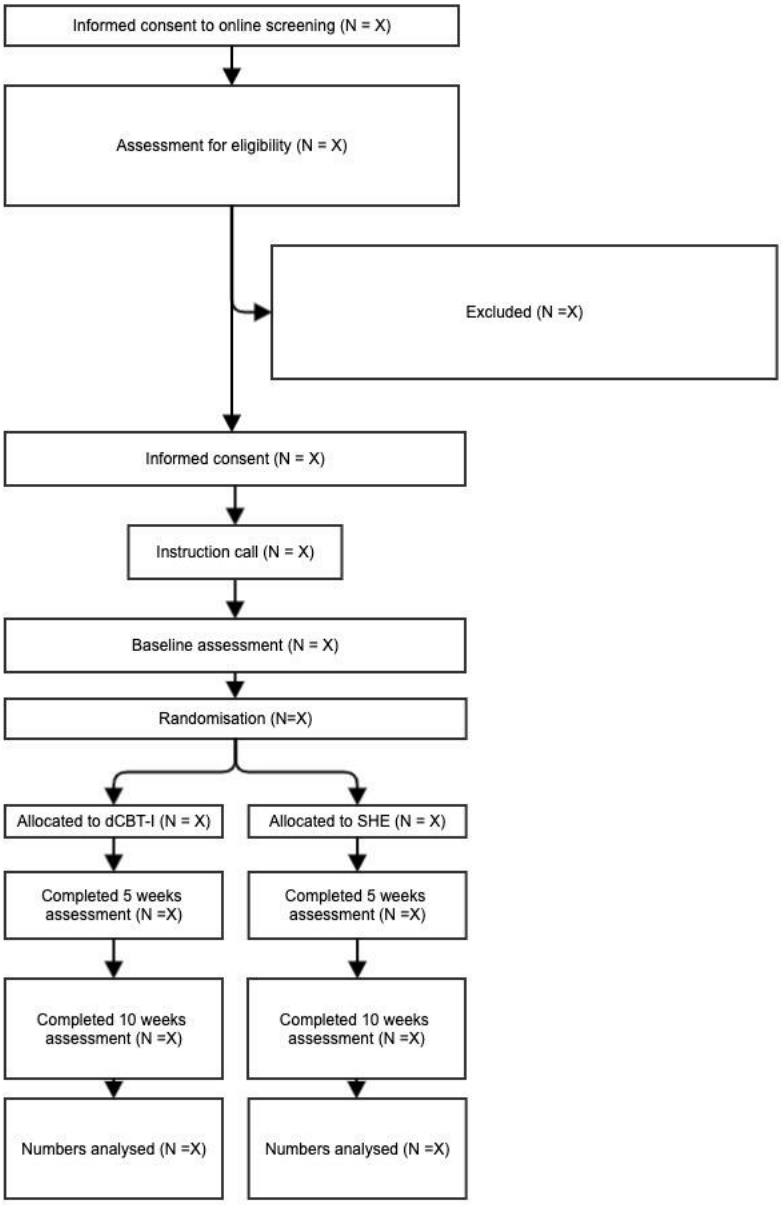

Frequencies (with percentages) for binary and categorical variables and means (and standard deviations), or medians (with lower and upper quartiles) for continuous variables will be presented by intervention group as well as overall.

Characteristics of participants

Baseline variables will be presented by randomised group using frequencies (with percentages) for binary and categorical variables and means (and standard deviations) or medians (with lower and upper

quartiles) for continuous variables. There will be no tests of statistical significance nor confidence intervals for differences between groups on any baseline variables. Baseline characteristics will be presented by randomised group and overall.

Definition of population for analysis

The primary analysis population will include all participants randomised who have at least one outcome measurement. Participants who withdraw from the trial will be included in the analysis until the point at which they withdraw. Participants will be analysed according to their allocated treatment group irrespective of what treatment they actually receive.

Primary analysis

Primary outcome

Our primary outcome measure will be the Facial Expression Recognition Task [FERT]. The FERT assesses the interpretation of facial expressions (Harmer et al., 2009). Faces with six different basic emotions (happiness, fear, anger, disgust, sadness, surprise) are displayed on a screen and participants are required to indicate the expression on the face by selecting a corresponding button. The task takes approximately 15-20 minutes to complete and assesses the following dependent variables: Emotion Accuracy, Emotion Misclassifications, Emotion Reaction time, Target Sensitivity for each emotion and Response bias for each emotion. The primary outcomes for the study are accuracy for sad and happy facial expression and are defined as the percentage of accurately identified faces for each emotion (Number of correct responses for each emotion)/(Number of faces of each emotion). We will also report Emotion Misclassification and Emotion Reaction Time (see exploratory analyses, below).

The main efficacy analysis will be conducted on a modified intention-to-treat basis including all participants having at least one outcome. The primary analyses (emotional processing of faces) differences between treatment groups (dCBT-I and SHE), will be estimated with mixed linear regression models. The main objective of the statistical analyses is to assess the effect of dCBT-I on the primary outcome at the end of the intervention period, 10 weeks. To this end a linear mixed modelling will be employed. In such models, the continuous outcome variable measured at the post randomisation time points features as the dependent, the baseline value of the outcome, stratification factors (sex, age, depression severity and insomnia severity), treatment arm (dCBT-I or SHE), assessment time (5 or 10 weeks) and a treatment x time interaction term included as covariates. To account for correlation between repeated measures on the same individual a subject-varying random intercept will be included. Time will be considered as a categorical variable and model fitted assessed for inclusion of a random slope. Model assumptions will be checked using diagnostic plots. Modelling will be based on the assumption that data are missing at random and will be fitted using restricted maximum likelihood estimation. Results from all analyses will be summarised at 5 and 10 weeks with 2 sided 97.5 % CIs and we will present the data from both adjusted (including stratification variables) and unadjusted models. The adjusted model will be considered the primary analysis.

Handling missing data

The analysis will use statistical techniques for handling missing outcome data under a missing-at random assumption (MAR). The availability of the outcome data for the primary outcome at 10 weeks will be summarised by randomised group.

The mixed effects model implicitly accounts for data missing at random, however the data missingness mechanism will be explored. Logistic regression models will explore any association between baseline characteristics and availability of the primary outcome at 10 weeks. Missing primary outcome data will be reported overall and by randomised group. Covariates found to be predictive of missingness ($P < 0.05$) will be included in the analysis model in a sensitivity analysis of the primary outcome.

If post randomisation variables, such as compliance with treatment, or baseline variables are found to be predictive of non-completion of outcome, such that it would not be suitable to adjust for them in the main analyses, then multiple imputation will be considered.

Handling outliers

Any outliers will be checked and verified to ensure that they are true values. Outliers will be identified as those observations more than four standard deviations from the mean. Once they have been confirmed, a sensitivity analysis will be carried out to assess the impact of these values on the results by excluding these participants (see below).

Multiple comparisons and multiplicity

The primary outcome is clearly stated in the protocol. Due to the use of 2 primary outcomes (accuracy for sad and happy faces, respectively) 0.05/2 = 0.025 will be considered significant for the main analysis.

Model assumptions

Standard residual diagnostics, such as inspection of the histogram of the residuals, will be used to assess the appropriateness of the model. If assumptions are violated we will consider firstly using the change in accuracy from baseline as the dependent variable. If the assumptions are still violated we will consider alternative non- parametric approaches.

Secondary analysis

Secondary outcomes

Continuous secondary outcomes will be analysed in the same way as the primary outcome. We will test the hypothesis for between-group difference in the outcomes at 5 weeks and 10 weeks using mixed effects models with baseline outcome measure, treatment assignment, assessment time, stratification variables and a treatment by time interaction as fixed effects with a subject-varying random intercept. We will apply standard regression diagnostics. The proposed analysis assumes that these secondary outcomes are continuous and satisfy the assumptions of the mixed effects model. Where these assumptions are not satisfied, the data will be transformed or if a transformation is not possible, a non-parametric approach to analysing the data will be adopted.

The secondary outcomes include:

Self-rated

The Difficulties in Emotion Regulation Scale (DERS), total score

The Patient Health Questionnaire (PHQ9)

The Penn State Worry Questionnaire PSWQ-Past Week (The PSWQ-PW)

The Perseverative Thinking Questionnaire (PTQ)

Insomnia Severity Index (ISI)

Mood Instability (one self-rated item from the Structured Clinical Interview for DSM-IV (SCID-II))

Munich Chronotype Questionnaire (MCTQ)

Positive and Negative Affect Schedule – Short Form (PANAS-SF)

Objective task performance

Emotion accuracy from the Facial Expression Recognition Task [FERT, see above] at week 5 will be considered a secondary outcome.

Emotional Categorisation Task [ECAT]. The ECAT assesses speed to respond to positive and negative self-referent personality descriptors. Participants are asked whether they would like or dislike to be referred to as each characteristic. The task takes approximately 5 minutes to complete and generates the following dependent variables: % Accuracy of each condition (positive or negative words), % misses of each condition (positive or negative words) and mean reaction time for correct responses for each condition (positive or negative words). The mean reaction time for correct responses for positive vs negative words will be considered the main outcome for this task, but we will also report % accuracy and % of misses (see below).

Emotion Recognition Memory Task [EMEM]. The EMEM measures recognition memory for affective words. Participants are presented with a series of words comprising the pleasant and unpleasant personality words that were previously presented to them in the ECAT, and a set of previously unseen distracter words. For each word, participants are required to report whether they have previously seen the word. The task takes approximately 10 minutes to complete and % accuracy of positive compared to negative words will be considered the main outcome for the task. We will also report misclassifications of each condition (positive or negative words) and mean reaction time for correct responses for each condition (positive and negative words) (see below).

Treatment engagement

For participants in the CBT-I intervention arm the following information will be reported: Total number of completed sessions in Sleepio.

For participants in the sleep hygiene arm the following information will be reported: Number of unique IP addresses logging in to the bespoke Sleep Hygiene advice page.

Mediation analysis

The following variables will be investigated as possible mediators (at week 5) of the association between treatment group and depressive symptoms (at week 10):

Changes in recognition accuracy for happy and sad facial expressions (FERT)

Change in reaction times for positive compared with negative words (ECAT)

Change in self-rated emotion regulation, worry and preservative thinking (reductions in DERS, PSWQ and PTQ scores)

Change in level of morningness (MSF SC from MCTQ)

Change in mood (PANAS-SF, positive and negative affect)

If the efficacy analysis shows significant between-group differences in these measures at 5 and 10 weeks, modern causal inference methods will be used to investigate the mediation hypotheses. Parametric regression models will be employed to test for the indirect effect of emotion regulation/processing/morningness/mood at week 5 on depression severity (PHQ-9) at 10 weeks. Since all the measures are continuous, the indirect effects are calculated by multiplying relevant pathways and bootstrapping is used to produce valid standard errors for the indirect effects. Results will be presented as indirect effects with 95% bootstrapped percentile CIs and also as percentage of the total effect that is mediated. All analyses will adjust for baseline measures of the mediators. An adjusted model, including the stratification variables will also be presented. Mediation analyses are potentially biased by measurement error in mediators and hidden confounding between mediators and outcomes and we will investigate the sensitivity of the estimates to these problems.

CACE analysis

A complier-average causal effect (CACE) analysis of the primary outcome will be carried out to determine what effect the level of compliance to the intervention has on the treatment effect i.e. an estimate of the treatment effect amongst compliant patients. Descriptive statistics will be presented for the primary outcome in the control group, and in the intervention group split by the number of Sleepio sessions attended (i.e. 0,1,2,3, 4, 5, 6).

Baseline characteristic of the participants by compliance status will be reported. Statistical testing of baseline characteristics with compliance will be carried out to determine factors associated with compliance. A table of baseline characteristics by control, complier and non-complier with statistical comparison between compliers and non-compliers in the CBT-I intervention arm will be reported.

To obtain an unbiased estimate of the effect of compliance to the intervention on treatment effect, we will produce the complier-average causal effect estimates of the difference between the mean accuracy scores for sad and happy faces (FERT) for the compliers in the Sleepio group compared to the would be

compliers in the control group (CACE). For the primary CACE analysis, a sufficient level of adherence will be defined as completing at least 3 Sleepio sessions. The analysis will be performed using a two stage residual inclusion regression with randomisation as an IV. In the first stage, a regression of treatment receipt on randomisation will be conducted. In the second stage, a regression of the outcome on treatment receipt will be conducted, adjusting for the first-stage residual as covariates, as well as the same covariates as the intention-to-treat analysis. Bootstrapping will be used to obtain CI due to the two stage approach.

Exploratory analysis

Exploratory analyses will be conducted to assess the effect of dCBT-I on other expressions in the FERT (fear, anger, disgust, surprise, neutral), including the grouping of positive (happy, surprise) vs negative (fear, anger, disgust) expressions. We will also report and conduct exploratory analyses on reaction times and emotion misclassifications from the FERT. Emotion Misclassification is defined as the percentage of misclassifications for each emotion (Number of responses to each emotion when that emotion not displayed/Number of faces of all other expressions). Emotion reaction time is the calculated mean reaction time for correct responses for each emotion (if no correct responses, recorded as a missing value).

For the secondary objective tasks (ECAT, EMEM), we will further report and analyse % accuracy and % of misses (ECAT) and misclassifications of each condition (positive or negative words) and mean reaction time for correct responses for each condition (positive and negative words) (EMEM).

We will report sleep diary data collected as part of the dCBT-I treatment (in the intervention group) and also report % of participants scoring < 11 on the ISI and < 10 on the PHQ-9 after treatment, to provide a clinical context.

Possible predictors of treatment effects on the FERT will be analysed and reported. Specifically, we will investigate early/late chronotype, self-rated emotion regulation, high vs low depression scores (PHQ-9 dichotomised at 15) and worry as predictors of responses. Moderators will be assessed separately by repeating the primary analysis model and including an interaction term between randomised treatment and the moderator.

Sensitivity analysis

A sensitivity analysis will be carried out to assess the impact of outliers. As explained above, outliers will be identified as those observations more than four standard deviations from the mean. A sensitivity analysis will be carried out to assess the impact of these values on the results by excluding these participants.

References

Carney CE, Harris AL, Moss TG, et al. (2010) Distinguishing rumination from worry in clinical insomnia. *Behaviour Research and Therapy* 48(6). Elsevier Ltd: 540–546. DOI: 10.1016/j.brat.2010.03.004.

Hom MA, Stanley IH, Rogers ML, et al. (2016) The association between sleep disturbances and depression among firefighters: emotion dysregulation as an explanatory factor. *Journal of Clinical Sleep Medicine* 12(2): 235–245. DOI: 10.5664/jcsm.5492.

Krause AJ, Simon E Ben, Mander BA, et al. (2017) The sleep-deprived human brain. *Nature Reviews Neuroscience* 18(7). Nature Publishing Group: 404–418. DOI: 10.1038/nrn.2017.55.

Liu DY and Thompson RJ (2017) Selection and implementation of emotion regulation strategies in major depressive disorder: An integrative review. *Clinical Psychology Review* 57. Elsevier Inc.: 183–194. DOI: 10.1016/j.cpr.2017.07.004.

Mauss IB, Troy AS and LeBourgeois MK (2013) Poorer sleep quality is associated with lower emotion-regulation ability in a laboratory paradigm. *Cogn Emot* 27(3): 567–576. DOI: 10.1080/02699931.2012.727783.

Morin CM and Benca R (2012) Chronic insomnia. *The Lancet* 379(9821). Elsevier Ltd: 1129–1141. DOI: 10.1016/S0140-6736(11)60750-2.

Morin CM, Drake CL, Harvey AG, et al. (2015) Insomnia disorder. *Nature Reviews Disease Primers* 1. Nat Rev Dis Primers: 15026. DOI: 10.1038/nrdp.2015.26.

Reddy R, Palmer CA, Jackson C, et al. (2017) Impact of sleep restriction versus idealized sleep on emotional experience, reactivity and regulation in healthy adolescents. *Journal of Sleep Research* 26(4): 516–525. DOI: 10.1111/jsr.12484.

Tamm, Sandra, Nilsonne G, Schwarz J, et al. (2019) Sleep restriction caused impaired emotional regulation without detectable brain activation changes-A functional magnetic resonance imaging study. *Royal Society Open Science* 6(3). DOI: 10.1098/rsos.181704.

Wilkinson PO, Croudace TJ and Goodyer IM (2013) Rumination, anxiety, depressive symptoms and subsequent depression in adolescents at risk for psychopathology: A longitudinal cohort study. *BMC Psychiatry* 13. DOI: 10.1186/1471-244X-13-250.
