# Supplementary material for: Emotional Processing Following Digital Cognitive Behavioral Therapy for Insomnia in People With Depressive Symptoms: A Randomized Clinical Trial
Source: JAMA Netw Open. 2025 Feb 27;8(2):e2461502. doi: 10.1001/jamanetworkopen.2024.61502 (PMC11868973; doi:10.1001/jamanetworkopen.2024.61502)
Supplement: Supplement 2. — eTable 1. List of Reasons for Online Screening Exclusion eTable 2. Reasons for Withdrawal From the Study eTable 3. Odds Ratio of the Risk of Insomnia and Depression eTable 4. Missing Data on the Primary Outcome (FERT) eTable 5. Outcome Missingness on the Primary Outcome (FERT) at 10 Weeks eTable 6. Sensitivity Analyses of the Primary Outcome at 10 Weeks eTable 7. Total Number of Completed Sessions in Sleepio From the Treatment Group eTable 8. Baseline Characteristics of Complier (Completed ≥3 Sessions) vs Non-complier (Completed <3 Sessions) in the Treatment Group eTable 9. Primary Outcomes of Complier vs Non-Complier in the Treatment Group eTable 10. Complier Average Causal Effect (CACE) Analysis eTable 11. Means and Standard Deviation on Tertiary Outcomes Across Timepoints eTable 12. Subgroup Analysis of the Primary Outcomes (FERT) at 10 Weeks eTable 13. Descriptive Data on Suicidal Ideation, Mood Instability, and Treatment Engagement eFigure. Standardised Effects of CBT-I on Depressive Symptoms Week 10) in Mediation Models with Mediators (Week 5), Including Emotional Regulation Difficulties (A), Worry (B), Positive Affect (C), and Negative Affect (D) eTable 14. Sleepio Content per Session eTable 15. The Breakdown of Sleep Restriction Therapy Procedures (Session 3-5) Within Sleepio eMethods. Data Cleaning on MCTQ Variables [file jamanetwopen-e2461502-s002.pdf]

Supplementary Online Content

Tamm S, Tse KYK, Hellier J, et al. The effect of digital cognitive behavioral therapy for insomnia on emotional processing in individuals with depressive symptoms. *JAMA Netw Open*. 2025;8(2):e2461502. doi:10.1001/jamanetworkopen.2024.61502

eTable 1. List of *R*easons for *Online Screening Exclusion*

eTable 2. Reasons for Withdrawal From the Study

eTable 3. Odds Ratio of the Risk of Insomnia and Depression

eTable 4. Missing Data on the Primary Outcome (FERT)

eTable 5. Outcome Missingness on the Primary Outcome (FERT) at 10 Weeks

eTable 6. Sensitivity Analyses of the Primary Outcome at 10 Weeks

eTable 7. Total Number of Completed Sessions in Sleepio From the Treatment Group

eTable 8. Baseline Characteristics of Complier (Completed ≥ 3 Sessions) vs Non-complier (Completed < 3 Sessions) in the Treatment Group

eTable 9. Primary Outcomes of Complier vs Non-Complier in the Treatment Group

eTable 10. Complier Average Causal Effect (CACE) Analysis

eTable 11. Means and Standard Deviation on Tertiary Outcomes Across Timepoints

eTable 12. Subgroup Analysis of the Primary Outcomes (FERT) at 10 Weeks

eTable 13. Descriptive Data on Suicidal Ideation, Mood Instability, and Treatment Engagement

eFigure. Standardised Effects of CBT-I on Depressive Symptoms Week 10) in Mediation Models with Mediators (Week 5), Including Emotional Regulation Difficulties (A), Worry (B), Positive Affect (C), and Negative Affect (D)

eTable 14. Sleepio Content per Session

eTable 15. The Breakdown of Sleep Restriction Therapy Procedures (Session 3-5) Within Sleepio

eMethods. Data Cleaning on MCTQ Variables

This supplementary material has been provided by the authors to give readers additional information about their work.

© 2025 Tamm S et al. *JAMA Network Open.*

eTable 1. List of reasons for online screening exclusion

Reasons for Online Screening Exclusion	n
No Consent	6
Age	56
Not living in the UK	7
English	2
No Internet Access	2
Alcohol	21
Shift Worker	210
PHQ-9 score <10	145
Did not meet SCI criteria	295
Possible Restless Leg Syndrome	245
Possible Narcolepsy	231
Possible Parasomnias	8
Possible Circadian Rhythm Disorder (Evening / Morning)	79
Possible Apnoea	102
Antiepileptic Drugs	5
Concurrent Psychological Therapy for insomnia or depression	34
Sleeping Pills	88
Mental Health Medications	42
Previous Participation in Online Sleep Treatment	9
Epilepsy	1
Mania / Bipolar Disorder	2
Multiple Sclerosis	2
Parkinson's Disease	1
Other Neurological Disorder	7
Prognosis <6 months or Surgery	1
Recreational Drug Use	3
Suicidal thoughts	4
Incomplete screening response	1728

Screening ceased at the earliest sign of exclusion, so reasons listed above are not mutually exclusive.

© 2025 Tamm S et al. *JAMA Network Open.*

eTable 2. Reasons for withdrawal from the study

	n	
	CBT-I	SHE
**After randomisation**		
No improvement in sleep		2
Ill-health	1	
Investigator withdrawal for ineligibility (discovered participant with suicidal ideation at baseline)	1	

© 2025 Tamm S et al. *JAMA Network Open.*

eTable 3. Odds ratio of the risk of insomnia and depression

	No. of participants (%) [n]		Unadjusted odds ratio (95% CI)	Adjusted odds ratio [a] (95% CI)
	CBT-I	SHE		
**Scoring below clinical threshold for insomnia (ISI <11)**				
Week 5	27 (30.7) [88]	13 (13.8) [98]	2.89 (1.38, 6.06)	3.31 (1.52, 7.20)
Week 10	39 (50.0) [78]	16 (16.5) [97]	5.06 (2.52, 10.16)	5.58 (2.64, 11.81)
**Scoring below clinical threshold for depression (PHQ-9 <10)**				
Week 5	49 (55.7) [88]	31 (31.6) [98]	2.72 (1.49, 4.94)	3.30 (1.62, 6.74)
Week 10	48 (61.5) [78]	30 (30.9) [97]	3.57 (1.91, 6.69)	3.99 (1.94, 8.21)

[a] Adjusted for stratification variables: sex, age, baseline ISI and baseline PHQ9.

© 2025 Tamm S et al. *JAMA Network Open.*

eTable 4. Missing data on the primary outcome (FERT)

	Number of missing data (%)			
	CBT-I	SHE	Odds ratio (95% *CI*)	*p*
**Week 5**	10 (10.0%)	6 (5.8%)	1.79 (0.63, 5.57)	0.277
**Week 10**	22 (22.0%)	7 (6.7%)	3.83 (1.61, 10.24)	0.002

© 2025 Tamm S et al. *JAMA Network Open.*

eTable 5. Outcome missingness on the primary outcome (FERT) at 10 weeks

Baseline characteristics	Predict missingness, p	CBT-I Missing data at W10 (n = 22)	CBT-I Complete data at W10 (n = 78)	SHE Missing data at W10 (n = 7)	SHE Complete data at W10 (n = 97)
**Age, mean (SD) in years**	0.829	48.1 (10.0)	50.2 (10.3)	51.3 (12.0)	48.6 (9.9)
**Sex, n (%)**					
Female	0.816	17 (81.0%)	63 (80.8%)	6 (85.7%)	79 (81.4%)
Male		4 (18.2%)	15 (19.2%)	1 (14.3%)	18 (18.6%)
Prefer not to say		1 (4.5%)	0 (0.0%)	0 (0.0%)	0 (0.0%)
**Ethnicity, n (%)**					
White	0.195	19 (86.4%)	71 (91.0%)	6 (85.7%)	91 (93.8%)
Mixed/Multiple Ethnic		0 (0.0%)	1 (1.3%)	0 (0.0%)	4 (4.1%)
Asian or Asian British or Asian Scottish		1 (4.5%)	3 (3.8%)	1 (14.3%)	1 (1.0%)
Caribbean or Black		1 (4.5%)	0 (0.0%)	0 (0.0%)	0 (0.0%)
Other		0 (0.0%)	3 (3.8%)	0 (0.0%)	1 (1.0%)
Prefer not to say		1 (4.5%)	0 (0.0%)	0 (0.0%)	0 (0.0%)
**Employment status, n (%)**					
Employed (Full-time)	0.472	11 (50.0%)	28 (35.9%)	3 (42.9%)	44 (45.4%)
Employed (Part-time)		6 (27.3%)	26 (33.3%)	3 (42.9%)	33 (34.0%)
Unemployed		0 (0.0%)	6 (7.7%)	0 (0.0%)	5 (5.2%)
Retired		3 (13.6)	10 (12.8%)	1 (14.3%)	12 (12.4%)
Full time student		0 (0.0%)	3 (3.8%)	0 (0.0%)	1 (1.0%)
Full time homemaker or carer		2 (9.1%)	5 (6.4%)	0 (0.0%)	2 (2.1%)
**Highest level of qualification, n (%)**					
GCSE or equivalent	0.280	2 (9.1%)	4 (5.1%)	1 (14.3%)	6 (6.2%)
A level or equivalent		3 (13.6%)	3 (3.8%)	0 (0.0%)	6 (6.2%)
College		3 (13.6%)	10 (12.8%)	1 (14.3%)	14 (14.4%)
University Undergraduate		8 (36.4%)	36 (46.2%)	3 (42.9%)	38 (39.2%)
University Postgraduate		6 (27.3%)	25 (32.1%)	2 (28.6%)	33 (34.0%)

Baseline Clinical Outcomes	Predict missingness, p	CBT-I Missing data at W10 (n = 22)	CBT-I Complete data at W10 (n = 78)	SHE Missing data at W10 (n = 7)	SHE Complete data at W10 (n = 97)
**Insomnia severity (ISI), mean (SD)**					
Total score	0.553	20.00 (3.41)	19.15 (3.55)	18.43 (3.36)	19.21 (3.85)
**Depression, PHQ-9, mean (SD)**					
Total score	0.676	14.55 (4.07)	13.58 (3.92)	14.57 (1.62)	14.69 (4.64)
**Positive and Negative Affect, PANAS, mean (SD)**					
Positive Affect	0.011	24.82 (8.41)	20.97 (6.94)	21.86 (8.67)	20.31 (6.10)
Negative Affect	0.066	27.86 (8.47)	24.51 (6.91)	29.71 (5.82)	26.38 (7.66)
**Emotional Regulation Difficulties, DERS, mean (SD)**					

© 2025 Tamm S et al. *JAMA Network Open.*

Total score	0.852	95.86 (21.58)	94.41 (22.90)	100.57 (17.05)	100.61 (22.64)
**Worry, PSWQ, mean (SD)**					
Total score	0.148	56.55 (18.06)	61.82 (14.39)	65.57 (12.00)	63.75 (13.36)
**Perseverative Thinking, PTQ, mean (SD)**					
Total score	0.731	37.45 (11.32)	36.81 (9.03)	39.00 (6.06)	39.82 (9.42)
**Chronotype, MCTQ, mean (SD) [n]**					
Midpoint of sleep period on work-free days, sleep corrected (HH:MM)	0.345	03:10 (63.41)	03:15 (79.21)	02:49 (78.04)	03:29 (81.72)
Social jetlag (mins)	0.206	49.87 (51.74)	30.38 (33.14)	15.33 (24.76)	32.85 (34.90)
**Emotional Categorisation Task, ECAT, mean (SD)**					
Reaction time: positive words (millisecond)	0.555	1473.41 (234.77)	1461.08 (217.21)	1452.71 (252.70)	1428.20 (208.92)
Reaction time: negative words (millisecond)	0.173	1518.68 (279.93)	1461.62 (202.37)	1496.29 (203.42)	1448.07 (212.88)
**Emotion Recognition Memory Task, EMEM, mean (SD)**					
Accuracy: positive words (%)	0.639	75.80 (12.13)	75.13 (9.59)	73.21 (10.18)	73.32 (11.98)
Accuracy: negative words (%)	0.634	77.73 (11.67)	76.79 (10.23)	75.00 (12.25)	75.31 (12.37)

© 2025 Tamm S et al. *JAMA Network Open.*

eTable 6. Sensitivity analyses of the primary outcome at 10 weeks

	Unadjusted mean (SD/SE*) [n]		Adjusted difference (95% *CI*)	*p*
	CBT-I	SHE		
**Adjusted for baseline predictor of outcome missingness [a]**				
FERT Accuracy, Happy faces, W10 (%)	53.44 (16.77) [78]	51.39 (15.75) [97]	3.00 (-1.10, 7.10)	0.152
FERT Accuracy, Sad faces, W10 (%)	61.18 (13.77) [78]	63.35 (11.17) [97]	-0.62 (-3.58, 2.34)	0.683
**Multiple Imputation [b]**				
FERT Accuracy, Happy faces, W10 (%)	53.2 (1.90*) [100]	51.3 (1.67*) [104]	2.51 (-1.88, 6.90)	0.265
FERT Accuracy, Sad faces, W10 (%)	61.6 (1.34*) [100]	63.4 (1.17*) [104]	-0.78 (-3.99, 2.43)	0.635

	Unadjusted, mean (SD) [n]		Adjusted difference (97.5% CI)	*p*	Cohen's *d* (95% *CI*)
Assessment	CBT-I	SHE			
**Unadjusted model [c]**					
FERT Accuracy, Happy faces, W10 (%)	53.44 (16.77) [78]	51.39 (15.75) [97]	2.80 (-2.60, 8.20)	0.246 [d]	0.23 (-0.16, 0.61)
FERT Accuracy, Sad faces, W10 (%)	61.18 (13.77) [78]	63.35 (11.17) [97]	-2.61 (-6.46, 1.24)	0.131 [d]	-0.24 (-0.55, 0.07)

Abbreviations: CBT-I, digital Cognitive Behavioural Therapy for Insomnia; SHE, Sleep Hygiene Education; FERT, Facial Expression Recognition Task.

[a] Linear mixed effects model with an unstructured variance-covariance structure for random effects, modelled against baseline outcome measure, stratification variables (sex, age, baseline ISI score and baseline PHQ-9 score), additional baseline factors that predict missingness (baseline PANAS PA), treatment arm (CBT-I or SHE) and assessment timepoint (5 or 10 weeks), and an interaction between assessment timepoint and treatment arm as fixed effects; Participant as random effect.

[b] Multiple imputation was completed using *mice* package from R. The imputation model included treatment arm, stratification variables (sex, age, baseline ISI score and baseline PHQ-9 score), additional baseline factors that predict missingness (baseline PANAS PA). Missing values of the primary outcome were imputed by means of the predictive mean matching (PMM) method using chained equations. We generated ten imputed datasets and visually inspected the observed and imputed values using plots. We combined the parameter estimates according to Rubin's rule (Rubin, 1987).

Linear mixed effects model with an unstructured variance-covariance structure for random effects, modelled against baseline outcome measure, stratification variables (sex, age, baseline ISI score and baseline PHQ-9 score), treatment arm (CBT-I or SHE) and assessment timepoint (5 or 10 weeks), and an interaction between assessment timepoint and treatment arm as fixed effects; Participant as random effect.

[c] Unadjusted model included treatment, timepoint and the interaction of treatment and timepoint as fixed effects; Participant as random effect.

[d] Co-primary outcomes were presented as 97.5 CI; p-value < 0.025 is considered significant for co-primary outcomes.

© 2025 Tamm S et al. *JAMA Network Open.*

eTable 7. Total number of completed sessions in Sleepio from the treatment group

Completed session(s)	n (%)
1	84 (84%)
2	80 (80%)
3	68 (68%)
4	59 (59%)
5	55 (55%)
6	47 (47%)

© 2025 Tamm S et al. *JAMA Network Open.*

eTable 8. Baseline characteristics of complier (completed ≥ 3 sessions) vs non-complier (completed < 3 sessions) in the treatment group

Baseline characteristics	Predict adherence, p	CBT-I Non-complier [a] (n = 32)	CBT-I Complier [a] (n = 68)
**Age, mean (SD) in years**	0.301	48.2 (9.37)	50.5 (10.6)
**Sex, n (%)**			
Female	0.393	24 (75.0%)	56 (8.2%)
Male		7 (21.9%)	12 (17.7%)
Prefer not to say		1 (3.1%)	0 (0.0%)
**Ethnicity, n (%)**			
White	0.761	29 (90.6%)	61 (89.7%)
Mixed/Multiple Ethnic		0 (0.0%)	1 (1.5%)
Asian or Asian British or Asian Scottish		1 (3.1%)	3 (4.4%)
Caribbean or Black		1 (3.1%)	0 (0.0%)
Other		0 (0.0%)	3 (4.4%)
Prefer not to say		1 (3.1%)	0 (0.0%)
**Employment status, n (%)**			
Employed (Full-time)	0.049	17 (53.1%)	22 (32.4%)
Employed (Part-time)		8 (25.0%)	24 (35.3%)
Unemployed		1 (3.1%)	5 (7.3%)
Retired		4 (12.5%)	9 (13.2%)
Full time student		0 (0.0%)	3 (4.4%)
Full time homemaker or carer		2 (6.3%)	5 (7.4%)
**Highest level of qualification, n (%)**			
GCSE or equivalent	0.083	2 (6.3%)	4 (5.9%)
A level or equivalent		4 (12.5%)	2 (2.9%)
College		3 (9.4%)	10 (14.7%)
University Undergraduate		14 (43.8%)	30 (44.1%)
University Postgraduate		9 (28.1%)	22 (32.4%)

Baseline Clinical Outcomes	Predict adherence, p	CBT-I Non-complier [a] (n = 32)	CBT-I Complier [a] (n = 68)
**Insomnia severity (ISI), mean (SD)**			
Total score	0.581	19.63 (2.97)	19.21 (3.78)
**Depression, PHQ-9, mean (SD)**			
Total score	0.313	14.38 (3.95)	13.51 (3.96)
**Positive and Negative Affect, PANAS, mean (SD)**			
Positive Affect	0.265	23.03 (8.02)	21.25 (7.11)
Negative Affect	0.544	25.91 (8.38)	24.94 (6.89)
**Emotional Regulation Difficulties, DERS, mean (SD)**			
Total score	0.560	96.66 (20.73)	93.82 (23.40)
**Worry, PSWQ, mean (SD)**			
Total score	0.105	57.03 (18.16)	62.37 (13.62)

© 2025 Tamm S et al. *JAMA Network Open.*

**Perseverative Thinking, PTQ, mean (SD)**			
Total score	0.426	38.06 (11.07)	36.43 (8.74)
**Chronotype, MCTQ, mean (SD) [n]**			
Midpoint of sleep period on work-free days, sleep corrected (HH:MM)	0.387	03:25 (64.37)	03:09 (81.52)
Social jetlag (mins)	0.675	36.81 (44.29)	33.21 (35.36)
**Emotional Categorisation Task, ECAT, mean (SD)**			
Reaction time: positive words (millisecond)	0.805	1455.81 (225.98)	1467.54 (218.78)
Reaction time: negative words (millisecond)	0.547	1493.72 (244.37)	1464.97 (211.10)
**Emotion Recognition Memory Task, EMEM, mean (SD)**			
Accuracy: positive words (%)	0.305	73.75 (11.27)	75.99 (9.57)
Accuracy: negative words (%)	0.903	77.19 (11.07)	76.91 (10.32)

[a] Complier is defined as those who completed ≥ 3 sessions in Sleepio; Non-complier is defined as those who completed < 3 sessions in Sleepio

© 2025 Tamm S et al. *JAMA Network Open.*

eTable 9. Primary outcomes of complier vs non-complier in the treatment group

	Mean (*SD*) [n]		
	Compliers in dCBT-I	Non-compliers in dCBT-I	All in dCBT-I
Facial Expression Recognition, FERT, Accuracy - Happy faces at W10 (%)	53.62 (17.30) [64]	52.58 (14.63) [14]	53.44 (16.77) [78]
Facial Expression Recognition, FERT, Accuracy - Sad faces at W10 (%)	62.16 (12.58) [64]	56.68 (18.17) [14]	61.18 (13.77) [78]

© 2025 Tamm S et al. *JAMA Network Open.*

eTable 10. Complier average causal effect (CACE) analysis

	Differences (95% *CI*)	*p*
**Facial Expression Recognition, FERT, Accuracy - happy faces at W10 (%)**		
ITT - Adjusted model [a]	3.01 (-1.09, 7.11)	0.150
CACE - Unadjusted model	2.50 (-3.39, 8.38)	0.408
CACE - Adjusted model [b]	4.35 (-2.56, 11.25)	0.220
**Facial Expression Recognition, FERT, Accuracy - sad faces at W10 (%)**		
ITT - Adjusted model [a]	-0.54 (-3.50, 2.42)	0.721
CACE - Unadjusted model	-2.65 (-7.19, 1.89)	0.253
CACE - Adjusted model [b]	-3.23 (-8.37, 1.91)	0.220
**Insomnia Severity, ISI at W10**		
ITT - Adjusted model [a]	-4.27 (-5.67, -2.87)	<0.001
CACE - Unadjusted model	-5.44 (-7.44, -3.51)	<0.001
CACE - Adjusted model [b]	-5.42 (-7.34, -3.51)	<0.001
**Depression Severity, PHQ-9 at W10**		
ITT - Adjusted model [a]	-3.91 (-5.20, -2.62)	<0.001
CACE - Unadjusted model	-5.43 (-7.41, -3.44)	<0.001
CACE - Adjusted model [b]	-4.63 (-6.34, -2.92)	<0.001

[a] Adjusted for baseline outcome measure and stratification variables (sex, age, baseline ISI and baseline PHQ9).
[b] Adjusted for baseline predictor of compliance (employment status), baseline outcome measure and stratification variables (sex, age, baseline ISI and baseline PHQ9).

© 2025 Tamm S et al. *JAMA Network Open.*

eTable 11. Means and standard deviation on tertiary outcomes across timepoints

Assessment	Unadjusted, mean (SD) [n] CBT-I	SHE	Adjusted difference (95% CI) [a]
**Sleep Quality, PSQI, Total sleep time (mins)**			
Week 5	338.18 (76.99) [88]	331.99 (82.46) [98]	15.4 (-2.14, 32.94)
Week 10	359.03 (76.38) [78]	337.27 (85.63) [97]	29.4 (11.43, 47.37)
**Sleep Quality, PSQI, Sleep onset latency (mins)**			
Week 5	31.48 (35.95) [88]	58.21 (48.67) [98]	-28.6 (-37.48, -19.72)
Week 10	31.75 (37.56) [78]	51.19 (40.09) [97]	-19.5 (-28.56, -10.44)
**Sleep Quality, PSQI, Sleep efficiency (%)**			
Week 5	69.98 (18.35) [88]	63.75 (18.36) [98]	6.55 (1.92, 11.18)
Week 10	72.06 (18.86) [78]	65.16 (16.36) [97]	7.17 (2.43, 11.91)
**Facial Expression Recognition, FERT, Accuracy - fear faces (%)**			
Week 0	47.47 (20.65) [100]	47.18 (18.34) [104]	
Week 5	49.36 (18.50) [88]	49.82 (17.48) [98]	0.08 (-3.86, 4.02)
Week 10	49.63 (19.58) [78]	52.90 (17.36) [97]	2.55 (-1.47, 6.57)
**Facial Expression Recognition, FERT, Accuracy - anger faces (%)**			
Week 0	61.65 (14.03) [100]	63.35 (12.49) [104]	
Week 5	57.07 (15.70) [88]	57.02 (14.81) [98]	-1.72 (-4.93, 1.49)
Week 10	62.29 (16.26) [78]	59.29 (15.99) [97]	-3.19 (-6.46, 0.08)
**Facial Expression Recognition, FERT, Accuracy - disgust faces (%)**			
Week 0	62.63 (12.82) [100]	62.25 (13.97) [104]	
Week 5	58.46 (16.02) [88]	59.14 (15.36) [98]	0.60 (-3.17, 4.36)
Week 10	60.33 (17.59) [78]	62.85 (13.03) [97]	3.76 (-0.08, 7.61)
**Facial Expression Recognition, FERT, Accuracy - surprise faces (%)**			
Week 0	67.75 (7.12) [100]	67.89 (9.24) [104]	
Week 5	61.22 (6.84) [88]	59.49 (9.74) [98]	-2.16 (-4.77, 0.45)
Week 10	59.03 (11.81) [78]	59.75 (10.50) [97]	0.36 (-2.31, 3.03)
**Facial Expression Recognition, FERT, Accuracy - neutral faces (%)**			
Week 0	74.19 (19.00) [100]	70.71 (19.74) [104]	
Week 5	77.41 (19.56) [88]	76.20 (19.89) [98]	0.37 (-4.38, 5.11)
Week 10	80.92 (19.82) [78]	81.48 (16.52) [97]	2.58 (-2.30, 7.46)
**Facial Expression Recognition, FERT, Accuracy - positive faces (%) [b]**			
Week 0	57.34 (7.81) [100]	57.87 (7.76) [104]	
Week 5	58.00 (9.07) [88]	55.14 (9.78) [98]	-3.40 (-5.85, -0.95)
Week 10	56.23 (11.83) [78]	55.59 (10.22) [97]	-1.41 (-3.92, 1.10)
**Facial Expression Recognition, FERT, Accuracy - negative faces (%) [c]**			
Week 0	60.11 (10.36) [100]	61.24 (10.11) [104]	
Week 5	57.52 (10.51) [88]	58.32 (9.96) [98]	-0.36 (-2.50, 1.78)
Week 10	58.38 (12.84) [78]	59.66 (10.20) [97]	0.77 (-1.41, 2.94)
**Facial Expression Recognition, FERT, Misclassification - happy faces (%) [d]**			
Week 0	0.90 (0.96) [100]	1.11 (1.40) [104]	
Week 5	1.85 (2.21) [88]	1.34 (1.91) [96]	-0.64 (-1.20, -0.08)
Week 10	1.84 (2.77) [77]	1.49 (1.62) [97]	-0.51 (-1.08, 0.06)
**Facial Expression Recognition, FERT, Misclassification - sad faces (%) [d]**			
Week 0	5.39 (4.51) [100]	6.43 (4.77) [104]	
Week 5	4.72 (3.65) [88]	6.30 (5.41) [98]	-1.19 (-2.30, -0.08)
Week 10	4.23 (3.56) [78]	5.09 (4.59) [97]	-0.28 (-1.42, 0.86)
**Facial Expression Recognition, FERT, Reaction time - happy faces (milliseconds)**			
Week 0	1853.71 (307.07) [100]	1842.37 (319.48) [104]	
Week 5	1813.39 (304.73) [88]	1826.64 (337.77) [98]	-13.1 (-83.27, 57.07)
Week 10	1768.64 (285.89) [78]	1781.39 (332.30) [97]	-19.3 (-91.43, 52.83)
**Facial Expression Recognition, FERT, Reaction time - sad faces (milliseconds)**			
Week 0	1709.53 (294.32) [100]	1648.33 (256.73) [104]	
Week 5	1626.32 (281.90) [88]	1608.07 (239.26) [98]	-17.7 (-76.70, 41.30)
Week 10	1585.04 (247.69) [78]	1568.36 (254.92) [97]	-12.2 (-73.16, 48.76)
**Emotional Categorisation, ECAT, Accuracy - positive words (%)**			
Week 0	92.70 (9.30) [100]	93.80 (7.49) [104]	

© 2025 Tamm S et al. *JAMA Network Open.*

Week 5	95.06 (6.04) [88]	93.88 (7.31) [98]	1.45 (-1.00, 3.90)
Week 10	95.13 (8.22) [78]	94.85 (11.85) [97]	0.41 (-2.12, 2.94)
**Emotional Categorisation, ECAT, Accuracy - negative words (%)**			
Week 0	95.20 (7.38) [100]	96.25 (4.96) [104]	
Week 5	95.40 (7.04) [88]	94.29 (8.31) [98]	1.43 (-1.44, 4.29)
Week 10	95.77 (13.07) [78]	95.31 (11.92) [97]	0.58 (-2.38, 3.54)
**Emotional Categorisation, ECAT, Misses - positive words (%)**			
Week 0	3.40 (6.51) [100]	3.27 (5.39) [104]	
Week 5	2.33 (4.55) [88]	2.45 (4.50) [98]	-0.10 (-2.18, 1.98)
Week 10	2.69 (6.96) [78]	2.84 (10.58) [97]	-0.18 (-2.32, 1.95)
**Emotional Categorisation, ECAT, Misses - negative words (%)**			
Week 0	2.90 (5.87) [100]	1.88 (3.13) [104]	
Week 5	2.78 (5.13) [88]	2.86 (4.97) [98]	-0.16 (-2.31, 2.00)
Week 10	2.37 (6.38) [78]	3.09 (11.07) [97]	-0.78 (-2.99, 1.44)
**Emotion Recognition Memory, EMEM, Misclassification - positive words (%)**			
Week 0	24.73 (10.14) [100]	26.01 (10.86) [104]	
Week 5	23.95 (13.15) [88]	24.03 (13.45) [98]	0.88 (-2.28, 4.03)
Week 10	27.31 (11.96) [78]	26.52 (11.12) [97]	1.84 (-1.41, 5.09)
**Emotion Recognition Memory, EMEM, Misclassification - negative words (%)**			
Week 0	23.00 (10.51) [100]	23.97 (11.22) [104]	
Week 5	24.74 (13.15) [88]	25.36 (13.52) [98]	-0.40 (-3.62, 2.81)
Week 10	29.71 (11.88) [78]	30.49 (11.24) [97]	-0.07 (-3.37, 3.22)
**Emotion Recognition Memory, EMEM, Reaction time - positive words (milliseconds)**			
Week 0	1580.32 (381.33) [100]	1509.42 (334.95) [104]	
Week 5	1449.10 (272.20) [88]	1483.02 (427.93) [98]	-78.00 (-164.44, 8.44)
Week 10	1444.40 (271.03) [78]	1456.46 (405.27) [97]	-54.40 (-143.38, 34.58)
**Emotion Recognition Memory, EMEM, Reaction time - negative words (milliseconds)**			
Week 0	1570.90 (348.41) [100]	1494.53 (306.16) [104]	
Week 5	1494.08 (298.82) [88]	1555.79 (634.55) [98]	-118.80 (-228.36, -9.24)
Week 10	1458.00 (264.09) [78]	1484.11 (388.97) [97]	-86.30 (-199.00, 26.40)

Abbreviations: CBT-I, digital Cognitive Behavioural Therapy for Insomnia; SHE, Sleep Hygiene Education; PSQI, Pittsburgh Sleep Quality Index; FERT, Facial Expression Recognition Task; ECAT, Emotional Categorisation Task; EMEM, Emotion Recognition Memory Task.
Trials with reaction time ≥ 5000ms were excluded from the overall mean.
[a] Adjusted for baseline outcome measure and stratification variables (sex, age, baseline ISI and baseline PHQ9).
[b] Positive faces in FERT included combining the recognition accuracy for surprise and happy faces.
[c] Negative faces in FERT included combining the recognition accuracy for sad, fear, anger and disgust faces.
[d] Misclassification is defined as the number of responses to each emotion when that emotion not displayed/210

© 2025 Tamm S et al. *JAMA Network Open.*

eTable 12. Subgroup analysis of the primary outcomes (FERT) at 10 weeks

Subgroup for recognition accuracy of happy faces	*N*	Adjusted mean [a] (95% *CI*)	*Test of interaction (p value)*
**PHQ-9 score at baseline**			
<15	112	-1.64 (-7.11, 3.83)	0.209
≥15	92	-4.96 (-11.25, 1.33)	
**DERS score at baseline**			
<99	102	-5.62 (-11.44, 0.20)	0.882
≥99	102	-0.30 (-6.18, 5.58)	
**PSWQ score at baseline**			
<62	97	-4.45 (-10.45, 1.55)	0.893
≥62	107	-2.41 (-8.07, 3.25)	
**MCTQ MSFsc score at baseline**			
< 03:15	72	-2.73 (-9.28, 3.82)	0.987
≥ 03:15	77	-6.52 (-13.05, 0.01)	

Subgroup for recognition accuracy of sad faces	*N*	Adjusted mean [a] (95% *CI*)	*Test of interaction (p value)*
**PHQ-9 score at baseline**			
<15	112	1.04 (-2.90, 4.98)	0.187
≥15	92	-0.03 (-4.56, 4.50)	
**DERS score at baseline**			
<99	102	-0.79 (-4.98, 3.40)	0.141
≥99	102	2.10 (-2.15, 6.35)	
**PSWQ score at baseline**			
<62	97	0.79 (-3.52, 5.10)	0.588
≥62	107	-0.31 (-4.42, 3.81)	
**MCTQ MSFsc score at baseline**			
< 03:15	72	-1.93 (-6.38, 2.52)	0.821
≥ 03:15	77	2.96 (-1.59, 7.51)	

Abbreviations: PHQ-9, Patient Health Questionnaire-9; DERS, Difficulties in Emotion Regulation Scale; PSWQ, Penn State Worry Questionnaire; MCTQ MSFsc, Munich Chronotype Questionnaire, midpoint of sleep (sleep corrected).

[a] Linear mixed effects model with an unstructured variance-covariance structure for random effects, modelled against baseline outcome measure, stratification variables (sex, age, baseline ISI score and baseline PHQ-9 score), treatment arm (CBT-I or SHE) and assessment timepoint (5 or 10 weeks), and a 3-way interaction between assessment timepoint, treatment arm and moderator as fixed effects; Participant as random effect.

PHQ-9: the clinical cut-off for moderately severe depressive symptoms (pre-defined in the protocol).
DERS: the cut-off was based on median split.
PSWQ-past week: the cut-off was based on median split.
MCTQ MSFsc: the cut-off was based on median split.

© 2025 Tamm S et al. *JAMA Network Open.*

eTable 13. Descriptive data on suicidal ideation, mood instability, and treatment engagement

	Number of participants (%)	
	dCBT-I	SHE
**Sudden mood changes over the past week**		
Week 0	45 (45.0%)	46 (44.2%)
Week 5	27 (30.7%)	39 (39.8%)
Week 10	20 (25.6%)	36 (37.1%)
**Suicidal ideation (PHQ-9, item 9*)**		
Week 0	13 (13.0%)	18 (17.3%)
Week 5	9 (10.2%)	23 (23.5%)
Week 10	6 (7.7%)	23 (23.7%)
**Taken any medication for sleep problems**		
Week 5	9 (10.2)	4 (4.1%)
Week 10	7 (9.0%)	9 (9.3)
**Engaged with additional psychological therapies for sleep problems**		
Week 5	5 (5.7%)	0 (0%)
Week 10	0 (0%)	2 (2.1%)
**Received treatment for mental health**		
Week 5	7 (8.0%)	7 (7.1%)
Week 10	8 (10.3)	8 (8.2%)

* PHQ-9, item 9: "Thoughts that you would be better off dead or of hurting yourself in some way". Presence of suicidal ideation is defined by answering, "several days", "more than half the days", or "nearly every day".

© 2025 Tamm S et al. *JAMA Network Open.*

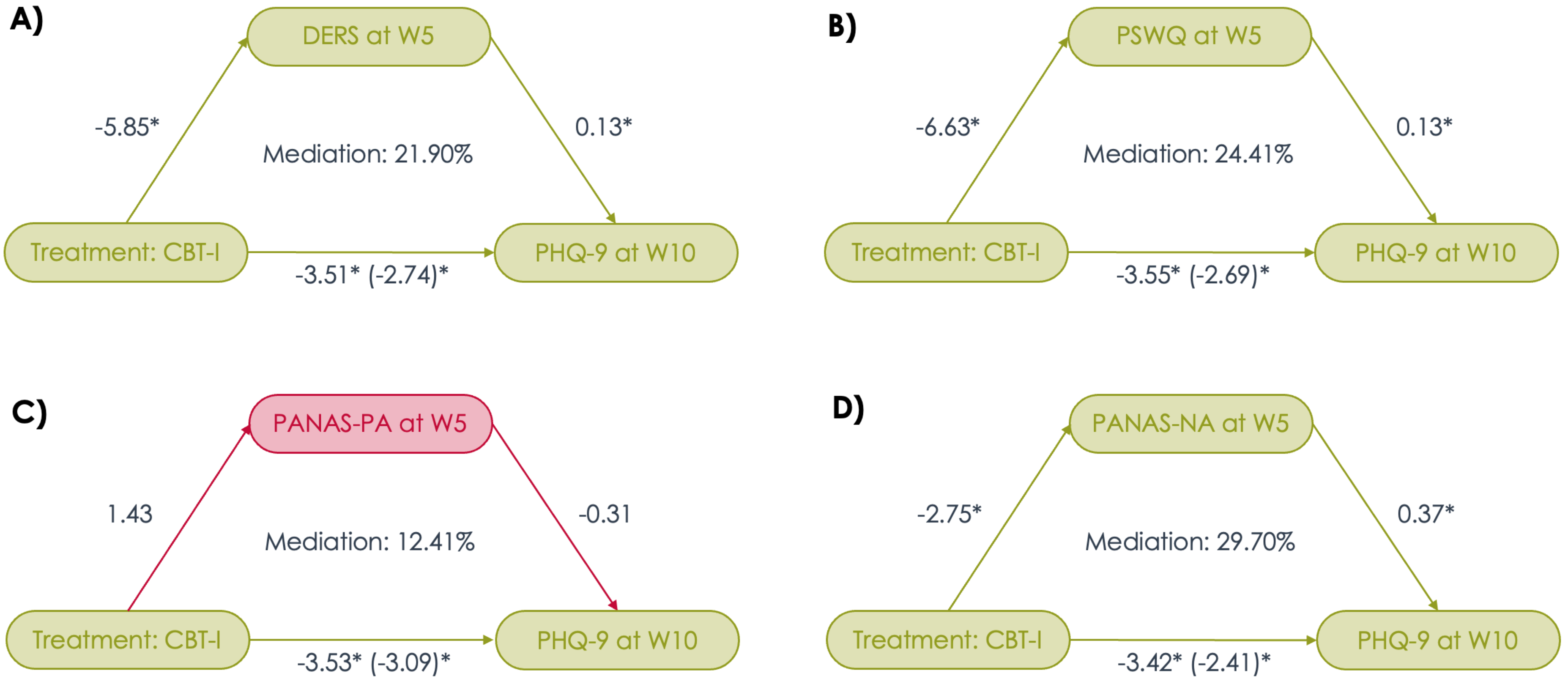

eFigure. Standardised Effects of CBT-I on Depressive Symptoms (week 10) in Mediation Models with Mediators (week 5), including Emotional Regulation Difficulties (A), Worry (B), Positive Affect (C), and Negative Affect (D). Green arrow represents significant path and red arrow represents non-significant path.

© 2025 Tamm S et al. *JAMA Network Open.*

eTable 14. Sleepio content per session

Session	Information
1	Formulation, goal setting, diary keeping, motivational contract
2	Sleep hygiene (lifestyle & bedroom), progressive relaxation, thought checker
3	Sleep hygiene (schedule), stimulus control, sleep restriction therapy
4	Adjustment to sleep window for sleep restriction therapy, continuation of cognitive therapy techniques, plus any of the followings (depending on priorities): Cognitive restructure, autogenic training, imagery, mindfulness, paradoxical intention
5	Adjustment to sleep window for sleep restriction therapy, continuation of cognitive therapy techniques, review goals, reinforce motivation
6	Course summary, review progress, instructions on how to move forward with everything learned

© 2025 Tamm S et al. *JAMA Network Open.*

eTable 15. The breakdown of sleep restriction therapy procedures (session 3-5) within Sleepio

SRT procedures	Sleepio's SRT criteria
Sleep window generation	Self-reported TST over last 2 weeks (sleep diary)
Minimum TIB	5 or 6 hours depending on general health, risk conditions, mental and physical health, age, type of employment and daytime sleepiness levels
SE criteria & change to sleep window	SE ≥ 90%, or having ≥ 20% increase in SE, or user indicates "struggling" or "feeling like quitting" => TIB + 15 min
Position of sleep window	User preference

© 2025 Tamm S et al. *JAMA Network Open.*

eMethods. Data Cleaning on MCTQ Variables

During data cleaning of the MCTQ variables, we noticed that a small proportion of participants may have answered in 12-hour format, rather than 24-hour format. We did self-evident corrections for participants whose rise-time rendered the bedtime nonsensical. We selected participants who reported bedtime between 06:00-15:00 and compared their MCTQ answers to their PSQI answers completed at the same timepoint. If their PSQI answers suggested they wrongly answered in 12-hour format for MCTQ, their answers were transformed into 24-hour format (W0, *n* = 27; W5, *n* = 21; W10, *n* = 24).

We also noticed a small proportion of participants produced conflicting answers for bedtime, sleep onset latency and wake time (e.g. bedtime + SOL > waketime). We selected participants who reported a sleep onset latency of 6+ hours and compared their MCTQ answers to PSQI answers completed at the same timepoint. If there was a large discrepancy between their MCTQ and PSQI answers (e.g. 25 mins SOL in PSQI vs. 7 hours 20 mins SOL in MCTQ), their answers were removed. We also removed participants who produced conflicting answers for bedtime, sleep onset latency and wake time (e.g. goes to bed at 3am, starts preparing to sleep at 7am, takes 4 hours to fall asleep and wakes up at 9am.; W0, *n* = 4; W5, *n* = 0; W10, *n* = 1).

© 2025 Tamm S et al. *JAMA Network Open.*
