# Supplementary material for: Emotional Processing Following Digital Cognitive Behavioral Therapy for Insomnia in People With Depressive Symptoms: A Randomized Clinical Trial
Source: JAMA Netw Open. 2025 Feb 27;8(2):e2461502. doi: 10.1001/jamanetworkopen.2024.61502 (PMC11868973; doi:10.1001/jamanetworkopen.2024.61502)
Supplement: Supplement 3. — Data Sharing Statement [file jamanetwopen-e2461502-s003.pdf]

Data Sharing Statement

Tamm. Emotional Processing Following Digital Cognitive Behavioral Therapy for Insomnia in People With Depressive Symptoms. *JAMA Netw Open*. Published February 25, 2025.
doi:10.1001/jamanetworkopen.2024.61502

Data
Additional Information: ISRCTN17117237
Data available: Yes
Data types: Deidentified participant data, Data dictionary
How to access data: corresponding author: simon.kyle@ndcn.ox.ac.uk
When available: With publication

Supporting Documents
Document types: None

Additional Information
Who can access the data: Researchers whose proposed use of the data has been approved
Types of analyses: De-identified participant data will be available in anonymised form from the corresponding author (SDK) on reasonable request (including a study outline), subject to review.
Mechanisms of data availability: After approval of a proposal
Any additional restrictions: N/A
